# High Phosphate Induces and Klotho Attenuates Kidney Epithelial Senescence and Fibrosis

**DOI:** 10.3389/fphar.2020.01273

**Published:** 2020-08-20

**Authors:** Jenny Maique, Brianna Flores, Mingjun Shi, Sierra Shepard, Zhiyong Zhou, Shirely Yan, Orson W. Moe, Ming Chang Hu

**Affiliations:** ^1^ Charles and Jane Pak Center for Mineral Metabolism and Clinical Research, University of Texas Southwestern Medical Center, Dallas, TX, United States; ^2^ Departments of Pathology, University of Texas Southwestern Medical Center, Dallas, TX, United States; ^3^ Department of Internal Medicine, University of Texas Southwestern Medical Center, Dallas, TX, United States; ^4^ Department of Physiology, University of Texas Southwestern Medical Center, Dallas, TX, United States

**Keywords:** aging, fibrosis, Klotho, p16, p21, PAI-1, phosphate, cellular senescence

## Abstract

Cellular senescence is an irreversible cell growth arrest and is associated with aging and age-related diseases. High plasma phosphate (Pi) and deficiency of Klotho contribute to aging and kidney fibrosis, a pathological feature in the aging kidney and chronic kidney disease. This study examined the interactive role of Pi and Klotho in kidney senescence and fibrosis. Homozygous Klotho hypomorphic mice had high plasma Pi, undetectable Klotho in plasma and kidney, high senescence with massive collagen accumulation in kidney tubules, and fibrin deposits in peritubular capillaries. To examine the Pi effect on kidney senescence, a high (2%) Pi diet was given to wild-type mice. One week of high dietary Pi mildly increased plasma Pi, and upregulated kidney p16/p21 expression, but did not significantly decrease Klotho. Two weeks of high Pi intake led to increase in plasminogen activator inhibitor (PAI)-1, and decrease in kidney Klotho, but still without detectable increase in kidney fibrosis. More prolonged dietary Pi for 12 weeks exacerbated kidney senescence and fibrosis; more so in heterozygous Klotho hypomorphic mice compared to wild-type mice, and in mice with chronic kidney disease (CKD) on high Pi diet compared to CKD mice fed a normal Pi diet. In cultured kidney tubular cells, high Pi directly induced cellular senescence, injury and epithelial-mesenchymal transition, and enhanced H_2_O_2_-induced cellular senescence and injury, which were abrogated by Klotho. Fucoidan, a bioactive molecule with multiple biologic functions including senescence inhibition, blunted Pi-induced cellular senescence, oxidation, injury, epithelial-mesenchymal transition, and senescence-associated secretary phenotype. In conclusion, high Pi activates senescence through distinct but interconnected mechanisms: upregulating p16/p21 (early), and elevating plasminogen activator inhibitor-1 and downregulating Klotho (late). Klotho may be a promising agent to attenuate senescence and ameliorate age-associated, and Pi-induced kidney degeneration such as kidney fibrosis.

## Introduction

Cellular senescence (herein, simply termed senescence) is an irreversible cell-cycle arrest and serves as a defense mechanism to prevent cancer cell transformation, growth, and metastasis (Childs et al., 2015; Sturmlechner et al., 2017; Mchugh and Gil, 2018; Ovadya and Krizhanovsky, 2018; Calcinotto et al., 2019; Docherty et al., 2019). In addition, senescence plays an important role in permanently restricting the propagation of damaged and defective cells during aging. Like autophagy and apoptosis, an appropriate senescence activity is critical for maintaining tissue integrity and function (White and Lowe, 2009).

Aging is an inevitable multi-organ deterioration initiated and accelerated by multiple factors. We have shown that Klotho deficiency and excess phosphate (Pi) have detrimental effects on aging (Fernandez et al., 2018; Shi et al., 2020). Compelling observational and animal studies showed that phosphotoxicity is associated with reduced longevity in several species (Kuro-O, 2010; Shi et al., 2020) and causes severely abnormal metabolism and ill effects on bone, kidney, cardiovasculature, and other organs in chronic kidney disease (CKD) (Kestenbaum et al., 2005; Tonelli et al., 2005; John et al., 2011; Calvo et al., 2019). However, the molecular mechanisms by which Pi mediates acceleration of aging, and initation and excerbation of cardiovascular disease in CKD are complex, multifactorial, and not fully understood (Ohnishi and Razzaque, 2010; Osuka and Razzaque, 2012; Hu et al., 2015; Ritter and Slatopolsky, 2016). Furthermore, whether and how Pi modulates senescence is largely not understood.

Klotho was serendipitously discovered and labeled an anti-aging gene, because homozygous Klotho hypomorphic mice demonstrated a premature aging-like syndrome whose manifestations include short lifespan, disturbed mineral metabolism including hyperphosphatemia and multiple organ degeneration such as infertility, arteriosclerosis, cardiomyopathy, ectopic calcification, skin atrophy, osteoporosis, and emphysema (Kuro-O et al., 1997). Klotho also suppresses senescence induced by replication and oxidative stress in cultured cells (De Oliveira, 2006; Haruna et al., 2007; Kuro-O, 2008; Liu et al., 2011). Removal of some key intermediates of the senescence signaling pathway could rescue the phenotypes and lifespan in Klotho hypomorphic mice, which further supports that abnormal senescence is part of the detrimental effects of Klotho deficiency (Eren et al., 2014; Sato et al., 2015).

Kidney fibrosis is one of the hallmarks of the aged kidney and of CKD, and also a deleterious driver for CKD progression (Cruz et al., 2000; Torres and Leof, 2011; Yang and Fogo, 2014; Humphreys, 2018). We previously showed that high Pi decreases circulating and kidney Klotho and induces kidney fibrosis, while low Klotho increases plasma Pi and synergistically exacerbates kidney fibrosis induced by high Pi diet (Hu et al., 2015; Shi et al., 2020). However, whether kidney fibrosis associated with Klotho deficiency is mediated through modulation of senescence remains uncertain.

In this study, we examined *in vivo* and *in vitro* effects of Pi and Klotho on senescence and fibrosis, and explored the profile of changes in several key intermediates including CDK inhibitor p21 (p21), CDK4 inhibitor p16^INK4^ (p16), plasminogen activator inhibitor-1 (PAI-1), and senescence marker protein 30 (SMP30) in mice kidneys and cultured normal rat kidney (NRK) cells. Different levels of Klotho and extracellular Pi were achieved through (1) genetic manipulation of the Klotho gene, (2) changes in dietary Pi or (3) induction of CKD by unilateral nephrectomy plus contralateral ischemia-reperfusion injury followed by feeding high dietary Pi, which is a combined model of excess Pi and Klotho deficiency. Through exploration of the timeline of changes in the senescence molecules under the effect of Pi, Klotho, and senescence inhibitor, we are the first, to our best knowledge, to propose that Pi activates senescence and induces epithelial-mesenchymal transition (EMT), and that Klotho may protect kidney tubular cells from Pi toxicity as a senescence suppressor.

## Materials and Methods

### Murine Strains and Chronic Kidney Disease Model

All animal work was conducted strictly following the Guide for the Care and Use of Laboratory Animals by the National Institutes of Health and was approved by the Institutional Animal Care and Use Committee at the University of Texas Southwestern Medical Center. Wild type (*WT*) 129 S1/SVlmJ (129sv) mice were purchased from Jackson laboratory (Bar Harbor, ME, USA) and housed in a temperature-controlled room (22.0 ± 0.2°C) with a 12:12 h light-dark cycle and were given *ad libitum* access to tap water and allowed free access to standard rodent chow (Teklad 2016, Harlan, Madison, WI) unless stated otherwise. Usually 10- to 12-week-old mice with equal number of males and females were used unless specifically indicated. All other mouse lines shown below had been cross-mated with *WT* 129sv mice for more than 10 generations and genotyped with standard PCR protocols described in the previous publications. Heterozygous Klotho hypomorphic (*kl/+*) mouse (Hu et al., 2011), and transgenic mice with Klotho overexpression (*Tg-kl*) (Hu et al., 2011) were kindly provided by Dr. Makoto Kuro-o (Jichi Medical University, Tochigi, Japan). Homozygous Klotho hypomorphic (*kl/kl*) mice were generated by cross-mating male and female *kl/+* mice.

The CKD model was prepared with established methods (Hu et al., 2011; Hu et al., 2017). Two weeks after unilateral nephrectomy plus contralateral ischemia reperfusion, mice were fed normal or high Pi content chow for 12 weeks.

### High Phosphate Diet

High Pi rodent chow containing 2.0% inorganic phosphorus (w:w) was purchased from Harlan (Teklad 08020, Harlan, Madison, WI) and given to *WT* mice or *kl/+* mice with CKD to examine its effect on senescence, Pi metabolism and Klotho expression. Normal Pi rodent chow containing 0.4% inorganic phosphorus was also purchased Harlan (Teklad 2016; Harlan, Madison, WI).

### Blood and Kidney Samples Collection

At predetermined times, mice were anesthetized with isoflurane and blood samples were collected in heparinized tubes and centrifuged at 3,000×*g* for 5 min at 4°C for plasma separation. At terminal study, mice were sacrificed under anesthesia, and the kidneys were isolated and sliced. One slice was fixed with 4% paraformaldehyde and embedded in a paraffin block or Tissue-Tek^®^ optimum cutting temperature (O.C.T.) for histological and immunohistological studies respectively; the remaining kidney tissue was snap-frozen in liquid N_2_ and stored at −80°C until RNA or protein extraction.

### Blood Chemistry and Plasma Klotho Measurement

Plasma Pi was analyzed in Core laboratory at UT Southwestern Medical Center using a Vitros Chemistry Analyzer (Ortho-Clinical Diagnosis, Rochester, NY). Plasma creatinine concentrations were measured by the O’Brien Kidney Research Center at UT Southwestern Medical Center using a P/ACE MDQ Capillary Electrophoresis System and photodiode detector (Beckman-Coulter, Fullerton, CA) at 214 nm (Hu et al., 2017). Soluble Klotho was determined by immunoprecipitation-immunoblot as described (Hu et al., 2010).

### Senescence Markers

We examined the state of senescence in kidneys and cultured kidney cells through senescence associated-β-gal staining (SA-β-gal), and other markers of senescence including heterochromatin protein 1-beta (HP1β), phosphorylation of histone H2A variant H2AX at the C-terminus (γ-H2AX), senescence marker protein 30 (SMP30), CDK4 inhibitor p16^INK4^ (p16), CDK inhibitor p21 (p21), and Lamin B1 in the kidney with either immunoblot, immunohistochemistry and/or qPCR. HP1β is an established senescence marker (Kosar et al., 2011; Crowe et al., 2014) because it is involved in chromatin reorganization and formation of senescence-associated with heterochromatin foci (Kosar et al., 2011; Aird and Zhang, 2013; Crowe et al., 2014). Gamma-H2AX is frequently used as a marker of senescence (Lawless et al., 2010; Knoppert et al., 2019). Both p16 and p21 are well-known cell cycle regulators and involved in senescence activation (Satyanarayana and Rudolph, 2004; Yosef et al., 2017). Lamin B1 is a considered a senescence marker as its loss is associated with induction of senescence (Heng et al., 2013). SMP30 is originally identified as an aging marker and is now considered to be a senescence inhibitor (Yumura et al., 2006).

### Primary and Secondary Antibodies

The following antibodies were used for immunoblotting and/or immunohistochemistry: mouse monoclonal antibody against β-actin (Sigma-Aldrich, St. Louis, MO); mouse monoclonal anti-CD31 (ab24590) (Abcam, Cambridge, MA); rat monoclonal anti-mouse CD31 (550274) (BD Pharmingen, San Jose, CA); rabbit polyclonal anti-Collagen I (ab34710) (Abcam, Cambridge, MA); rabbit polyclonal anti-Collagen IV (AB756P) (Millipore, Temecula, CA); goat polyclonal anti-fibrin (Nordic-MUbio, Susteren, The Netherlands); rabbit monoclonal anti-phospho-histone H2AX (Ser 139) (also called γ-H2AX) (Cell Signaling Technologies, Danvers, MA); mouse monoclonal anti-heterochromatin Protein-1 beta (HP1β) (Chemicon; EMD Millpore Corporation, Temecula, CA); rabbit polyclonal anti-NaCl-cotransporter (NCC) (Chemicon; EMD Millpore Corporation); mouse monoclonal PAI-1 (Santa Cruz Biotechnology, Santa Cruz, CA); rat monoclonal anti-Klotho antibody (KM2076) (TransGenics, Kobe, Japan); rat monoclonal anti-Klotho antibody (KM2119) (TransGenics, Kobe, Japan); rabbit polyclonal anti-p16^INK4A^ (Proteintech, Rosemont, IL); mouse monoclonal anti-p21 (Millipore, Temecula, CA); mouse monoclonal antibody against α-SMA (Sigma-Aldrich, St. Louis, MO); goat polyclonal anti-SMP30 (Santa Cruz Biotechnology, Santa Cruz, CA); and goat polyclonal anti-THP (G-20) (Santa Cruz Biotechnology). Secondary antibodies coupled to FITC, Alexa Fluor-647, or -568, or -488 or Cy5 were purchased from Molecular Probes/Invitrogen (Molecular Probes Inc., Eugene, OR) for immunofluorescence staining.

The lectins used to identify kidney tubular segment markers were from Vector Laboratories (Burlingame, CA). Rhodamine labeled dolichos biflorus agglutinin (DBA) was used for the identification of the collecting duct, and fluorescein lotus tetragonolobus lectin (LTL) for the proximal tubules. DAPI (Sigma-Aldrich, St Louis, MO) was used to label the nuclei. In addition, we used THP antibody to identify the thick ascending limb of loop of Henle (TAL), and Klotho and NCC antibodies to identify the distal tubules (DT).

### Kidney Histopathology

Kidney tissues were fixed in 4% paraformaldehyde for 16 h at 4 °C, and 4 μm sections of paraffin embedded kidney tissues were stained with trichrome (TC) and picrosirius red (PSR) staining to evaluate kidney fibrosis. Both stained kidney sections were examined and photographed by two histopathologists blinded to the experimental protocol. Fibrotic score based on fibrotic area and intensity were quantified with ImageJ software with published methods (Shi et al., 2016; Shi et al., 2018a).

### Immunohistochemistry

Four μm sections of paraffin-embedded kidney or cryosections were used for immunohistochemistry following protocols previously published (Li et al., 2020; Shi et al., 2020). For non-nuclear protein staining, paraffin-embedded kidney sections were de-waxed and rehydrated, then heated in a microwave in 10 mM citrate buffer (pH 6.0) for 30 min (20 min in boiling conditions) to retrieve antigens. For nuclear protein staining (HP1β, γ-H2AX, and p16^INK4A^), paraffin-embedded kidney sections were incubated with 20 μg/ml proteinase K in the prepared buffer (50 mM Tris, 1 mM EDTA, and 0.05% Triton-X 100, pH 8.0) at 37°C for 5 min in a humidified chamber to retrieve antigens. After rinsing with PBS-Triton X-100 and 1X-PBS, the sections were blocked with 2% bovine serum albumin/normal animal serum for 1 h and incubated with primary antibodies overnight at 4°C in a humidified chamber. The following day, the sections were washed with Tris buffer saline-Tween and probed with secondary antibodies. Prior to mounting, autofluorescence was quenched with Vector^®^ TrueVIEW^®^ Autofluorescence Quenching Kit (SP-8400) (Vector Laboratories, Burlingame, CA).

All primary antibodies for immunohistochemistry were diluted in DAKO antibody diluent (S3022 Agilent, Santa Clara, CA). All stains were carried out in triplicate. Images were examined and acquired with Zeiss LSM 880 Confocal Microscope system (Jena, Germany).

### SA-β-Gal Staining in Kidney Sections

Senescence associated-β-gal (SA-β-gal) staining in kidney tissues was performed with the SA-β-gal staining Kit (Cell Signaling Technologies, Danver, MA) based on published methodology (Jin et al., 2019) with minor modifications. In brief, kidneys were fixed in 4% paraformaldehyde (PFA) and frozen sections were prepared. Sections were stained overnight at 37°C. After washes with phosphate-buffer saline (PBS), kidney sections were counterstained with eosin and imaged by bright-field microscopy.

### Cell Line and Cell Culture

Normal rat kidney (NRK) cells, a kidney tubule epithelial cell line, were used to test the effect of Pi, H_2_O_2_, Klotho, and fucoidan (a senescence inhibitor) on markers of senescence, cell injury, and epithelial-mesenchymal transition. The culture media were harvested for lactate dehydrogenase (LDH) measurement with LDH Cytotoxicity Assay Kit (LDH cytotoxicity detection kit (Clontech Laboratories, Mountain View, CA) to evaluate cell injury, and for 8-hydroxydeoxyguanosine (8-OHdG) measurement with DNA Damage (8-OHdG) ELISA kit (ImmunoChemistry Technologies, Bloomington, Minnesota, USA) to evaluate oxidative DNA damage following the manufacturer’s protocols. To examine inhibitory effect of senescence on Pi toxicity, Fucoidan was purchased from Sigma-Aldrich (Saint Louis, MO) and dissolved in PBS. The working concentration for cell culture is 40 nM. Fucoidan is a sulfated polysaccharide present in brown algae and brown seaweed (Atashrazm et al., 2015; Fitton et al., 2019), and has anti-oxidant, anti-inflammatory, anti-angiogenic, and anti-cancer activity (Atashrazm et al., 2015; Fitton et al., 2019). Recently Fucoidan was also shown to suppress senescence (Lee et al., 2015; Lee et al., 2018).

To study the effect of Pi on SA-β-gal signal, NRK cells were seeded on culture dishes. SA-β-gal was detected in cultured cells as described in literature (Dimri et al., 1995). Briefly, cells grown on coverslips were stained with the previously mentioned kit (Cell Signaling Technologies, Danver, MA). The Cells were then imaged using differential interference contrast Zeiss Axiovert 100 inverted microscope with a Jenoptik Gryphax Camera. Each experimental condition was performed in triplicate. A minimum of 5 pictures in each cultured cell condition were randomly taken.

### Immunoblotting

Total kidney lysates covering all kidney zones were prepared in radioimmunoprecipitation assay (RIPA) buffer containing freshly added cocktail protease inhibitors [complete, ethylenediaminetetraacetic acid (EDTA)-free protease inhibitor cocktail; Sigma-Aldrich, St. Louis, MO]. Cell lysates were also prepared with RIPA mentioned above. Protein lysates were subjected to SDS-PAGE as described previously (Li et al., 2020; Shi et al., 2020) and transferred to polyvinylidene fluoride membranes. After incubation with primary antibodies, the filters were incubated with horseradish-peroxidase conjugated species-specific secondary antibodies (Bio-Rad, Hercules, CA), followed by application of enhanced chemiluminescence substrate (Bio-Rad Laboratories Inc., Hercules, CA). Densitometric analyses were performed with Image J software.

### RNA Extraction, Reverse Transcription, and Quantitative Real-Time Polymerase Chain Reaction (qPCR)

Total RNAs from mouse kidneys or NRK cells were extracted with RNAeasy kit (Qiagen, Germantown, MD) according to the manufacturer’s protocol. Complimentary DNA (cDNA) was generated with oligo-dT primers using SuperScript III First Strand Synthesis System (Invitrogen, Carlsbad, CA) according to the manufacturer’s protocol. PCR was performed with universal SYBR Green I Dye protocol (Life Technologies Corporation, Carlsbad, CA) in The ABI PRISM^®^ 7000 Sequence Detection System (qPCR/RTPCR) (7000 SDS instrument, Foster City, CA) and each sample was run in triplicate. Data are expressed at an amplification number of 2^-ΔΔCt^ by normalization to *cyclophilin* and compared to controls. The primers utilized are listed below.

The qPCR primers used for mouse transcripts are shown in **Table 1** including *Lmnb1* (Heng et al., 2013), *SMP30* (Kondo et al., 2013), *α-SMA*, *CTGF*, *cyclophilin* (Hu et al., 2015; Shi et al., 2018b), *Klotho* (Shi et al., 2018b), *PAI-1* (Eren et al., 2014), *TNFα*, *TGFβ*, mouse *IL-1β*, and *IL-6* (Kawane et al., 2010). The qPCR primers used for rat transcripts are also shown in **Table 1** including *α-SMA*, *IL-1β*, *IL6*, *TNFα* (Khan et al., 2013), *PAI-1*, *TGFβ *(Lu et al., 2015), *NGAL*, and *cyclophilin* (Hu et al., 2010).

**Table 1:** Mouse and rat primers used for qPCR.

	Forward primers	Reverse primers
**Mouse**		
*Lmnb1*	5’-CAG ATT GCC CAG CTA GAA GC-3’	5’-CAT TGA TCT CCT CTT CAT AC-3’
* SMP30*	5’-GAG GCA GCC TGA TGC TGG TAA-3’	5’-GAG CTG CAG TTC ACC CTG CAT A-3’
*α-SMA*	5’-GAG AAG CCC AGC CAG TCG-3’	5’-CTC TTG CTC TGG GCT TCA-3’
*CTGF*	5’-CTG GAA GAC ACA TTT GGC CC-3’	5’-CAG AAG GTA TTG TCA TTG GT-3’
*cyclophilin*	5’-TGC TCT TTT CGC CGC TTG CT-3’	5’-TCT GCT GTC TTT GGA ACT TTG TCT G-3’
*Klotho*	5’-AAC CAG CCC CTT GAA GGG AC-3’	5’-TGC ACA TCC CAC AGA TAG AC-3’
*PAI-1*	5’-ACGC CTG GTG CTG GTG AAT GC-3’	5’-ACG GTG CTG CCA TCA GAC TTG TG-3’
*TNFα*	5’-CAC AGA AAG CAT GAT CCG CGA CGT-3’	5’-CGG CAG AGA GGA GGT TGA CTT TCT-3’
*TGFβ*	5’-GAC CGC AAC AAC GCC ATC TA-3’	5’;-GGC GTA TCA GTG GGG GTC AG-3’
*IL-1β*	5’-CCA GCT TCA AAT CTC ACA GCA G-3’	5’-CTT CTT TGG GTA TTG CTT GGG ATC-3’
*IL-6*	5’-TCC AGT TGC CTT CTT GGG AC-3	5’-GTA CTC CAG AAG ACC AGA GG-3’
**Rat**		
*NGAL*	5’-CCA GTT CGC CAT GGT ATT TT-3’	5’-CCT TGA GGC CCA GAG ACT-3’
*α-SMA*	5’-GAT CAC CAT CGG GAA TGA ACG C-3’	5’-CTT AGA AGC ATT TGC GGT GGA C-3’
*cyclophilin*	5’-GTC TCT TTT CGC CGC TTG CT-3’	5’-TCT GCT GTC TTT GGA ACT TTG TCT G-3’
*PAI-1*	5’-CCT TCC AGA GTC CCA TAC A-3’	5’-CTG GCT CTT TCC ACC TCT-3’
*TNFα*	5’-ACT GAA CTT CGG GGT GAT TG-3’	5’-GCT TGG TGG TTT GCT ACG AC-3’
*TGFβ*	5’-ATA CGC CTG AGT GGC TGT CT-3’	5’-TGG GAC TGA TCC CAT TGA TT-3’
*IL-1β*	5’-CAC CTT CTT TTC CTT CAT CTT TG-3’	5’-GTC GTT GCT TGT CTC TCC TTG TA-3’
*IL-6*	5’-TGA TGG ATG CTT CCA AAC TG-3’	5’-GAG CAT TGG AAG TTG GGG TA-3

### Statistical Analysis

Data are expressed as mean ± SD unless otherwise specified. Analysis was performed with SigmaPlot 13.0 software (Systat Software, Inc. San Jose, CA). As appropriate, statistical analysis was performed using unpaired Student-t-test, or one-way or two-way analysis of variance (ANOVA) followed by Student-Newman-Keuls *post hoc* test when applicable as specified. A value of P ≤ 0.05 was considered statistically significant.

## Results

### High Senescence in Kidney Tubules in Klotho-Deficient Mice (*kl/kl*)

**Figure 1:**
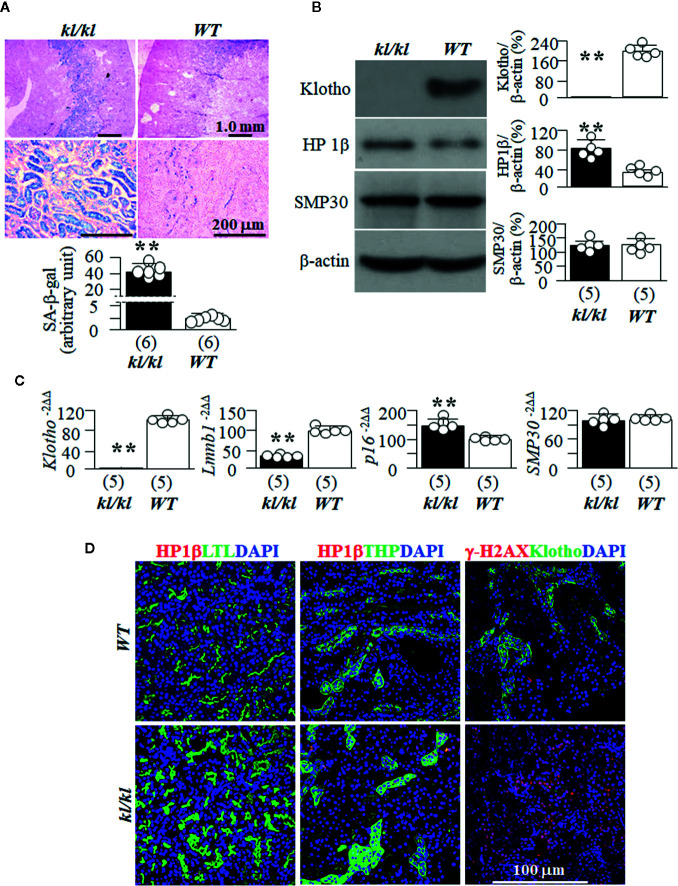
*WT* and *kl/kl* mice were given normal Pi diet and sacrificed at 6 weeks old. Each genotype had 6 mice. **(A)** SA-β-gal stain for the evaluation of cellular senescence; and Eosin counterstain in the kidneys of *kl/kl* and *WT* mice. Upper panel: representative images of SA-β-gal stain in kidney sections; bottom panel: quantitative score of SA-β-gal stain in kidney sections. **(B)** Immunoblots of total kidney lysates for Klotho and senescence markers. Left panel: representative blots for HP1β, SMP30 and β-actin (loading control) in total kidney lysates. Right panel: Summary of all immunoblots from each group. **(C)** Quantitative analysis of transcripts of *Klotho* and cellular senescence markers (*Lmnb1*, *p16*, and *SMP30*) in the kidneys. Sample number in each group is in brackets underneath each corresponding bar. Data are presented as mean ± S.D. with scatter plots of individual data points. **P<0.01 between two genotypes by un-paired Student t test for **(A–C)**. **(D)** Co-immunostaining for senescence markers (HP1β and γ-H2AX) (red color) with LTL (marker for PT, green), THP (marker for TAL, green), Klotho (marker for DT, green), and DAPI for nuclear stain (blue).

To explore if high senescence is associated with premature aging, we used homozygous Klotho hypomorphic mice (*kl/kl*) (Kuro-O et al., 1997) as an *in vivo* aging model, because this mouse strain has a short lifespan, undetectable Klotho in the circulation and kidney, and other multiple premature organ degeneration and dysfunctions resembling that were seen in aged animals (Kuro-O et al., 1997). We found higher levels of SA-β-gal staining (**Figure 1A**), a known marker of senescence (Dimri et al., 1995) in *kl/kl* mice compared to *WT* littermates. We then examined another two senescence markers HP1β and γ-H2AX, because HP1β is involved in chromatin reorganization and formation of senescence-associated with heterochromatin foci (Kosar et al., 2011; Aird and Zhang, 2013; Crowe et al., 2014), and γ-H2AX is frequently used as a marker of senescence (Lawless et al., 2010; Knoppert et al., 2019). We found higher levels of HP1β and absence of Klotho expression in the kidneys of *kl/kl* compared to *WT* mice (**Figure 1B**). In addition, we explored the changes in mRNA levels of Lamin B1 and p16, because loss of Lamin B1 is associated with induction of senescence (Heng et al., 2013), and activation of p16 with senescence activation (Satyanarayana and Rudolph, 2004). There were lower *Lmnb1*, higher *p16*, and no *Klotho* mRNA expression in the kidneys of *kl/kl* mice (**Figure 1C**), supporting that *kl/kl* mice had higher levels of senescence in the kidneys compared to *WT* mice at 6 weeks old as published (Eren et al., 2014; Sato et al., 2015). Finally we examined SMP30, as SMP30 loss is associated with senescence induction (Yumura et al., 2006). Interestingly and surprisingly, there was no alteration of SMP30 protein and mRNA, indicating that SMP30 may not be an intermediate to premature aging phenotypes in *kl/kl* mice (**Figures 1B, C** **)**.

### Tubular Segmental Distribution of Senescent Cells in the Kidney

SA-β-gal staining is prominently present in the cortex and outer medullary regions (**Figure 1A**) in both *WT* mice and *kl/kl* mice. This indicates that Klotho deficiency only enhances senescent magnitude, but does not alter SA-β-gal expression pattern. As HP1β staining provided convincing mapping of senescent signal in the kidneys of *WT* mice (**Figure 1D**), we co-stained HP1β with several well-established kidney tubular markers to map the profile of senescent cells in kidney tubular segments. Strong signal of HP1β was noted in both the proximal tubule (PT) stained with LTL and the thick ascending limb of loop of Henle (TAL) stained with THP, and γ-H2AX in distal tubules (DT) stained with Klotho (**Figure 1D**). There was a weak signal of HP1β in collecting ducts (CD) stained with DBA and in the distal tubules (DT) stained with Klotho or NCC in outer medullar zone of *WT* mice (**Supplemental Figure 1**). The expression pattern of HP1β is similar to the distribution of SA-β-gal staining in the kidneys of *WT* mice, suggesting that more senescent cells in PT and TAL may be one of reasons why those segments are more prone to kidney insults like ischemia-reperfusion induced AKI and nephrotoxins.

The weak signals of HP1β in the collecting ducts, in particular in inner medullar collecting ducts in the papilla (**Supplemental Figure 2**) suggests that SA-β-gal staining be not as sensitive as HP1β. In addition, more senescent cells in the inner medullar collecting ducts may be associated with medullary fibrosis (Farris et al., 2016) in certain scenarios such as hypertensive kidney damage (Haggitt et al., 1971) and chronic hypokalemia (Yalamanchili et al., 2018).

### Massive Fibrin Deposits and Tubulointerstitial Fibrosis in Klotho-Deficient Mice (*kl/kl*)

**Figure 2:**
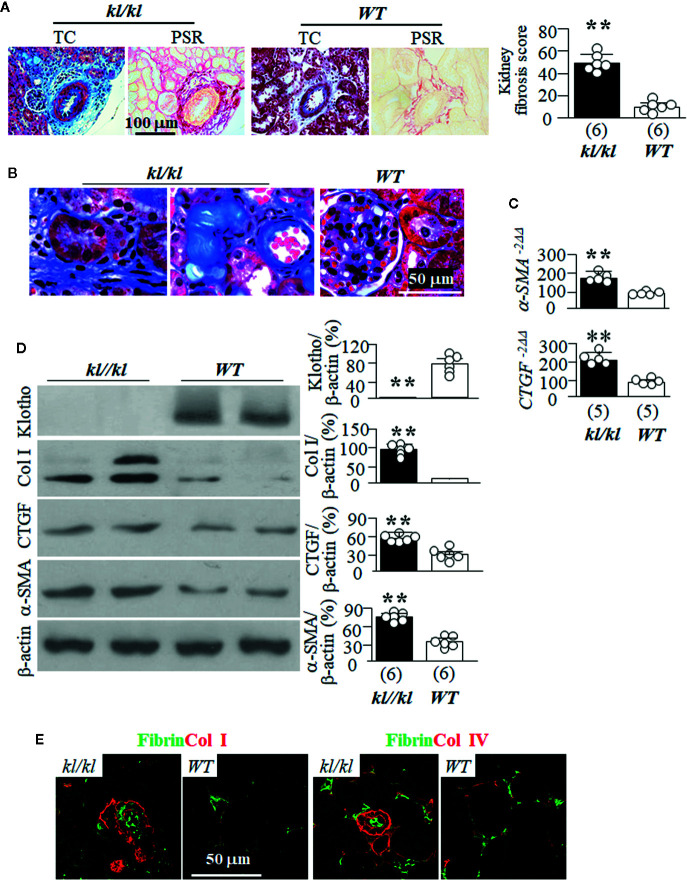
Massive tubulointerstitial fibrosis in *kl/kl* mice. *WT* and *kl/kl* mice were housed with normal Pi diet and sacrificed at 6 weeks old. Each genotype had 6 mice. **(A)** Kidney fibrosis evaluated with Trichrome (TC) stain and picro-sirius red (PSR) staining. Left panel: representative microscopic images; right panel: kidney fibrosis score. **(B)** Representative microscopic images of TC stain of kidneys of *WT* mice and *kl/kl* mice. **(C)** Quantitative analysis of transcripts of fibrotic markers (*a-SMA*, and *CTGF*) in the kidneys. **(D)** Immunoblots of total kidney lysates for Klotho and fibrotic markers. Left panel: representative blots for Klotho, Col I, CTGF, α-SMA and β-actin in total kidney lysates; right panel: summary of all immunoblots from each group. **(E)** The co-immunostaining for fibrin and collagen I (left panels) and collagen IV (right panel) in kidney sections. Sample number in each group is presented in bracket underneath corresponding bar. Data are presented as mean ± S.D. with scatter plots of individual data points. **P<0.01 between two genotypes by un-paired Student t test for **(C, D)**.

*kl/kl* mice have been shown to have more fibrosis in the kidney than in *WT* littermates (Sugiura et al., 2012). We used both Trichrome (TC) and picrosirius red staining (PSR) to map kidney fibrosis and found massive fibrosis in the tubulointerstitium and surrounding vessels of in the kidneys of *kl/kl* mice compared to *WT* mice (**Figures 2A, B**, and **Supplemental Figure 3**). Quantitative analysis of Klotho and some key fibrogenic markers with immunoblots showed lower expression of Klotho, and higher levels of Col I, CTGF, and α-SMA in the kidneys of *kl/kl* mice compared to *WT* mice (**Figures 2C, D**). Immunohistochemistry showed massive deposits of fibrin and Col I in the tubulointerstitium (**Figure 2E**, left panel), and fibrin and Col IV in the tubular basement membrane and lumen of kidney tubules as well as in peritubular capillary (**Figure 2E**, right panel). The higher expression of *α-SMA* and *CTGF* transcripts in the kidneys of *kl/kl* compared to *WT* mice supports the model of Klotho deficiency inducing kidney fibrosis. Interestingly, there were very limited areas overlapping fibrin and collagen I or IV in the kidneys, suggesting that fibrin and collagen may be deposited or accumulated in different compartments probably through distinct mechanisms.

**Figure 3:**
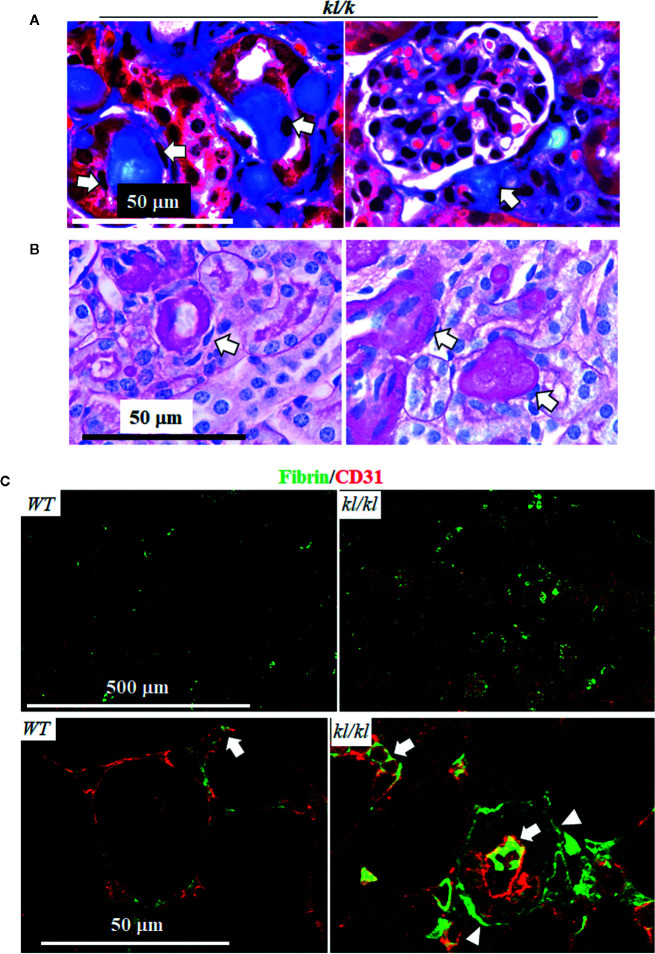
Massive fibrin accumulation in peritubular capillary (PTC) and interstitium in *kl/kl* mice. *WT* and *kl/kl* mice were fed normal Pi diet and sacrificed at 6 weeks old. Each genotype had 6 mice. Representative microscopic images of Trichrome (TC) stain **(A)** and of Periodic Acid–Schiff (PAS) **(B)** in kidney sections of *kl/kl* mice. Arrow depicts endothelial cells. **(C)** Representative microscopic images of co-immunostaining for fibrin (green) and CD31 (red) in kidney sections of *WT* and *kl/kl* mice. Upper panel: low magnification of microscopic images. Arrow depicts endothelial cells, and arrow head depicts kidney tubules.

Next, we examined the location of fibrin deposits in the kidney of *kl/kl* mice. In trichrome (**Figure 3A**) and PSR stained-kidney sections (**Figure 3B**), there were remarkable homogenous protein deposits along peritubular capillaries (depicted by arrows) in *kl/kl* mice (**Figures 3A, B**), but there were no such deposits in *WT* mice (data not shown). Double staining of fibrin and CD31, an endothelial marker, clearly illustrated scattered and tiny fibrin protein deposits in peritubular capillaries in *WT* mice (**Figure 3C** left panel). At the same time, the presence of fibrin in both the peritubular capillaries (depicted by arrows) as well as the kidney tubules (depicted by arrow heads) was both massive and disorganized in *kl/kl* mice (**Figure 3C**), suggesting ectopic fibrin deposits in the kidneys of *kl/kl* mice.

### High Pi Intake Induces More Kidney Tubular Senescence and Tubulointerstitial Fibrosis in *kl/+* Mice Than *WT* Mice

**Figure 4:**
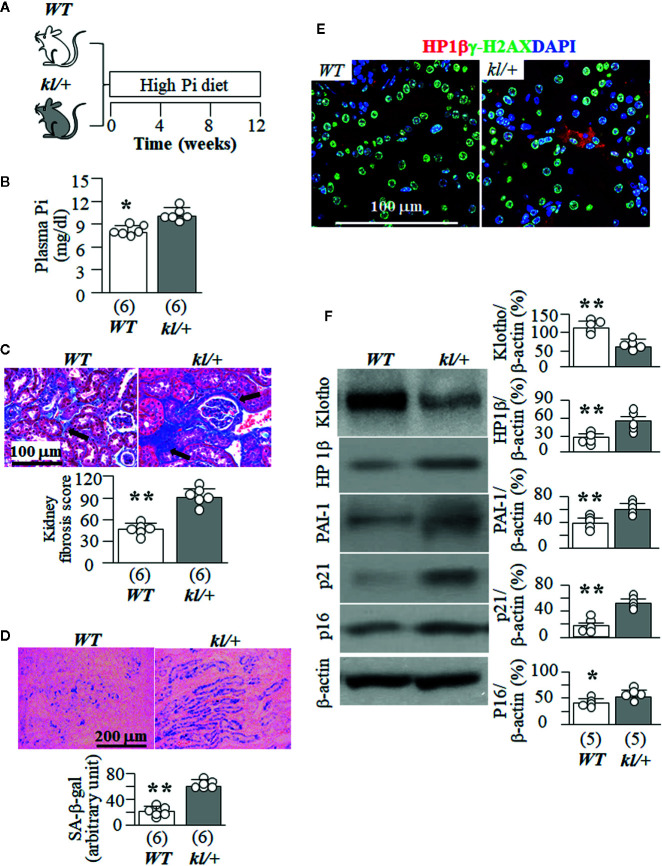
High Pi intake induces more senescence in kidney tubules and tubulointerstitial fibrosis in *kl/+* mice compared to *WT* mice. **(A)** *WT* and *kl/+* mice at 12 weeks old were fed with normal or high Pi diet for 12 weeks, and sacrificed under anesthesia. Each genotype had 6 mice. **(B)** Plasma Pi in *WT* and *kl/+* mice after 12-week dietary Pi intake. **(C)** Kidney fibrosis evaluated with TC staining. Upper panel: representative microscopic images; bottom panel: kidney fibrosis score. **(D)** SA-β-gal stain in kidney sections of *WT* and *kl/kl* mice. Upper panel: representative images of SA-β-gal stain in kidney sections; bottom panel: quantitative score of SA-β-gal stain in kidney sections. **(E)** Representative Immunohistochemistry for HP1β (red), γ-H2AX (green) and DAPI (blue) in kidney sections. Scale bar = 100 μm. **(F)** Immunoblots for Klotho and senescence markers in total kidney lysates. Left panel: representative immunoblots for Klotho, HP1β, PAI-1, p21, p16, and β-actin in total kidney lysates; right panel: summary of all immunoblots from each group. Sample number in each group is presented in bracket underneath corresponding bar. Data are presented as mean ± S.D. with scatter plots of individual data points. *P<0.05, **P<0.01 between two genotypes by un-paired Student t test.

Because *kl/kl* mice had extremely high plasma Pi (**Supplemental Figure 4**), and cannot tolerate high dietary Pi challenge, we next tested high Pi effect on kidney senescence in *WT* mice and the heterozygous Klotho deficient (*kl/+*) mice. *kl/+* mice had relatively lower Klotho levels in the circulation and kidneys than *WT* mice (Hu et al., 2010; Panesso et al., 2014; Shi et al., 2018b) but are relatively healthy at baseline without much kidney fibrosis and elevation of senescence markers in the kidney when they were fed normal Pi diet compared to *WT* mice (data not shown), and can withstand a high Pi diet for 12 weeks (**Figure 4A**). *kl/+* mice had higher plasma Pi (**Figure 4B**) and more kidney fibrosis (**Figure 4C**) compared with *WT* mice after a 12-week high Pi diet, implying that Klotho insufficiency exacerbates Pi retention, toxicity, and also directly worsens fibrosis in the kidney. Moreover, *kl/+* mice had higher plasma creatinine (0.16 ± 0.03 mg/dl) compared with *WT* mice (0.11 ± 0.02 mg/dl, p<0.05) after 12 weeks of high Pi diet. As shown in **Figure 4D**, a 12-week high Pi diet induced more SA-β-gal staining in the kidneys of *kl/+* mice compared with *WT* mice, indicating that Klotho deficiency accelerates high Pi-activated cellular senescence in the kidney. However, normal dietary Pi did not increase SA-β-gal stain in the kidney of *kl/+* mice, which was similar to *WT* mice when normal dietary Pi chow was given (**Supplemental Figure 5A**). Screening senescence markers in the kidneys further showed higher expression of HP1β and γ-H2AX (**Figures 4E, F**). As expected, much lower kidney Klotho protein and mRNA were noted in *kl/+* mice compared with *WT* mice after a 12-week Pi challenge (**Figure 4F**, and **Supplemental Figure 5B**). The changes in the transcripts of *Lmnb1*, *p16*, *α-SMA* and *CTGF* in the kidneys (**Supplemental Figure 5B**) supported that high Pi diet induced more senescence and fibrosis in *kl/+* mice compared to *WT* mice. However, whether high Pi directly induces cellular senescence or reduces kidney Klotho and consequently accelerates cellular senescence in the kidney is still inconclusive based on those *in vivo* results.

### High Pi Accelerates CKD Progression as Well as Senescence in CKD Mice

**Figure 5:**
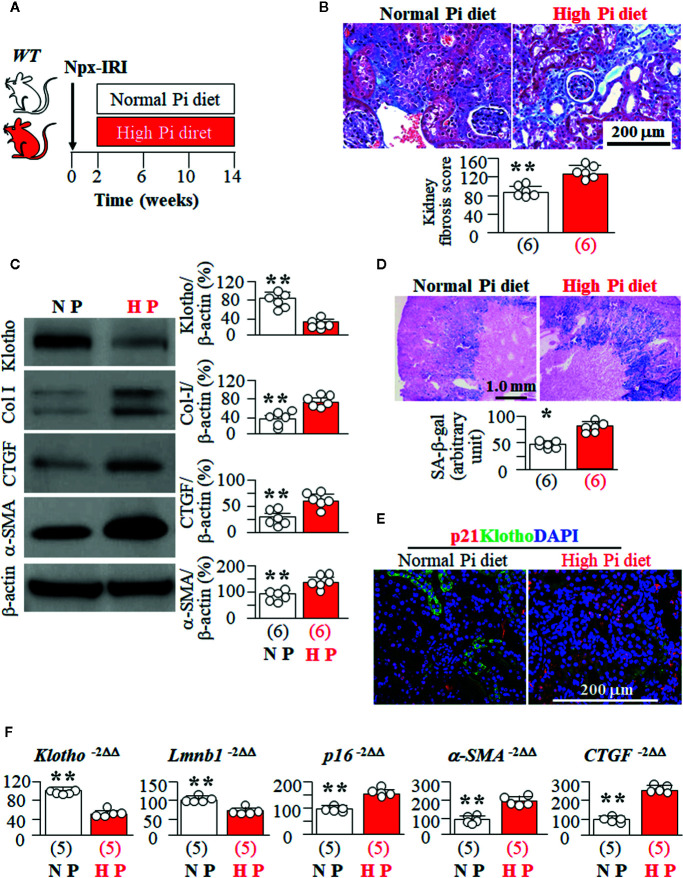
High Pi promotes senescence in the kidney and accelerates kidney deterioration in CKD mice. **(A)** *WT* mice at 12 weeks old underwent to CKD induction surgery and were fed with normal or high Pi diet for 12 weeks starting 2 weeks after induction of CKD. Each genotype had 6 mice. **(B)** Kidney fibrosis evaluated with TC staining. Upper panel: representative microscopic images; Bottom panel: kidney fibrosis score. **(C)** Immunoblots for Klotho and fibrotic markers in total kidney lysates. Left panel: representative immunoblots for Klotho, Col I, CTGF, α-SMA, and β-actin in total kidney lysates; right panel: summary of all immunoblots from each group. **(D)** SA-β-gal stain in kidney sections of CKD mice treated with normal or high Pi diet. Upper panel: Representative images of SA-β-gal stain in kidney sections; bottom panel: quantitative score of SA-β-gal stain in kidney sections. **(E)** Immunohistochemistry for HP1β (red), LTL (green), and DAPI (blue) in the kidney sections. **(F)** Quantitative analysis of transcripts of *Klotho*, senescent markers (*Lmnb1* and *p16*), and fibrotic markers (*a-SMA*, and *CTGF*) in the kidneys. Sample number in each group is presented in bracket underneath corresponding bar. Data represented as mean ± S.D. with scatter plots of individual data points. *P<0.05, **P<0.01 between two genotypes by un-paired Student t test for **(C, D, F)**.

High Pi has deleterious effects on CKD progression (Shi et al., 2016; Shi et al., 2020). To explore if high Pi enhances cellular senescence in CKD mice, we fed CKD mice with high vs. normal Pi diet (**Figure 5A**). As expected, there was an increase in plasma Cr (**Supplemental Figure 6A**), and kidney fibrosis (**Figures 5B, C**) along with elevation of plasma Pi (9.8 ± 0.8 mg/dl, n = 8) in CKD mice fed a high Pi diet compared to CKD mice fed a normal Pi diet (8.8 ± 0.6 mg/dl, n = 8, p < 0.05). In addition, CKD mice with high dietary Pi had lower Klotho expression and more SA-β-gal staining in the kidneys compared to CKD mice on normal dietary Pi (**Figures 5C, D**). Consistent with SA-β-gal staining, there was higher expression of HP1β, p21, and p16 proteins and mRNA, and lower expression of *Lmnb1* mRNA in the kidneys of CKD mice on a high Pi diet compared to CKD mice on a normal Pi diet (**Supplemental Figure 6B** and **Figures 5E, F**). This strongly indicates that high Pi exacerbates senescence in kidney tubules of CKD mice. There is higher expression of *α-SMA* and *CTGF* mRNA and lower expression of *Klotho* mRNA in the kidneys of CKD mice fed with high Pi diet than that in CKD mice fed with normal Pi diet (**Figure 5F**). Because lower Klotho protein expression was noted in the kidneys of CKD mice fed with high Pi diet compared to normal Pi diet, we are not able to decipher individual effect or synergistic effect of Pi and Klotho on cellular senescence in CKD mice. A cell culture model is needed to examine the individual and direct effects of Pi and Klotho on senescence.

### High Pi Induces Senescence and Promotes EMT in Cultured Kidney Tubular Cells

**Figure 6:**
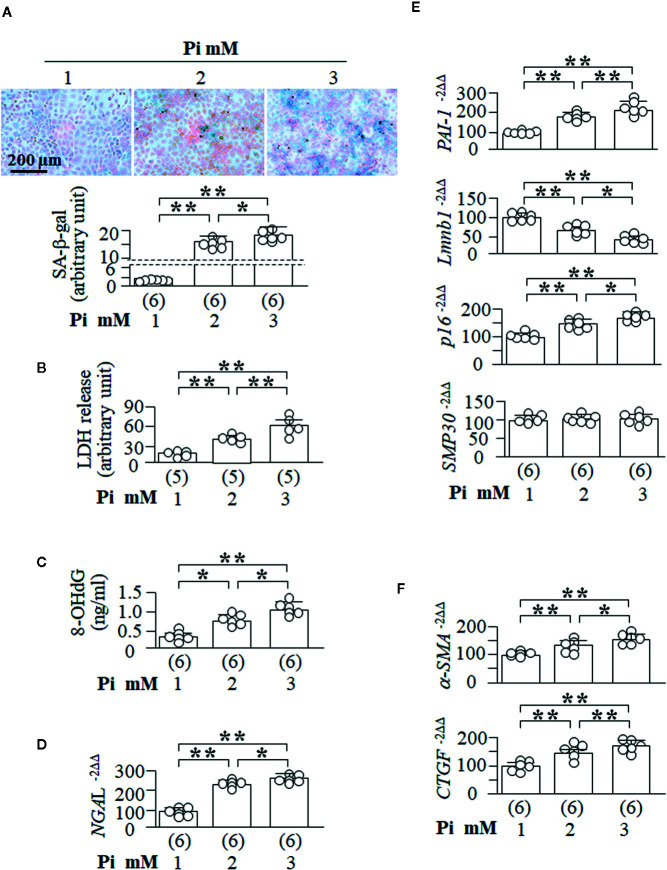
High ambient Pi induces senescence and increases fibrotic markers in cultured kidney tubular cells. NRK cells were seeded in 6-well plates and treated with Pi for 72 h. **(A)** SA-β-gal stain in the cells with eosin as contrast stain. Upper panel: representative images of SA-β-gal stain; bottom panel: quantitative score of SA-β-gal stain. **(B)** LDH and **(C)** 8-OHdg concentration in culture media collected from NRK cells. Quantitative analysis of transcripts of *NGAL*, kidney tubular cell injury markers **(D)**, senescence markers (*PAI-1*, *Lmnb1*, *p16*, and *SMP30*) **(E)**, and fibrotic markers (*a-SMA* and *CTGF*) **(F)** in NRK cells. Sample number in each group is presented in bracket underneath corresponding bar. Data are presented as mean ± S.D. with scatter plots of individual data points. *P<0.05, **P<0.01 between any two genotypes by one-way ANOVA followed by Student-Newman-Keuls *post hoc* test.

To explore if high Pi is able directly to induce cellular senescence, NRK cells were treated with different Pi concentrations in culture media. After 72 h of Pi treatment, SA-β-gal positive cells were significantly increased in a dose-dependent manner (**Figure 6A**). LDH concentration, a marker of cell injury, 8-OHdG levels in the culture media, a known marker of oxidation-induced DNA damage (Helbock et al., 1999; Ravikumar et al., 2014), and NGAL transcripts, another kidney tubular cell injury marker, were all significantly increased (**Figures 6B, D**) and comparable to an increase in the SA-β-gal staining score (**Figure 6A**) and the changes in senescence markers (decreased *Lmnb1* and elevated *PAI-1* and *p16*, but not *SMP30* mRNA) (**Figure 6E**). The changes in SA-β-gal, Lmnb1, PAI-1, and p16 were Pi-dose dependent.

It is well known that injured tubular epithelial cells exhibit abnormal cellular plasticity in that cells can dedifferentiate, re-enter the cell cycle, and subsequently mal-adapt to chronic and/or severe injury so as to change cell phenotype from epithelial cells to fibroblasts (Kalluri and Neilson, 2003; Liu, 2004). It is difficult to examine the fibrotic morphology in cultured cells as there is no histology, but exploration of fibrogenic markers in cultured cells is an alternative way to assess EMT. Notably, high Pi upregulated *α-SMA* and *CTGF* mRNA in NRK cells (**Figure 6F**), suggesting that high Pi induce EMT, consequently promoting fibrogenesis. Therefore, high Pi could directly induce senescence and cell injury, and initiate fibrosis.

To examine whether high Pi renders cells more vulnerable to oxidation, which takes place in acute kidney injury, we co-treated NRK cells with high Pi and H_2_O_2_, and found that high Pi amplified H_2_O_2_-induced senescence (**Supplemental Figure 7A**). Additionally, H_2_O_2_-induced elevation of LDH and 8-OHdG was amplified by high Pi at the dose-dependent manner (**Supplemental Figures 7B, C**). Therefore, high Pi synergistically increases H_2_O_2_-induced senescence and cell injury.

### Klotho Suppresses Senescence *In Vivo* and *In Vitro*

**Figure 7:**
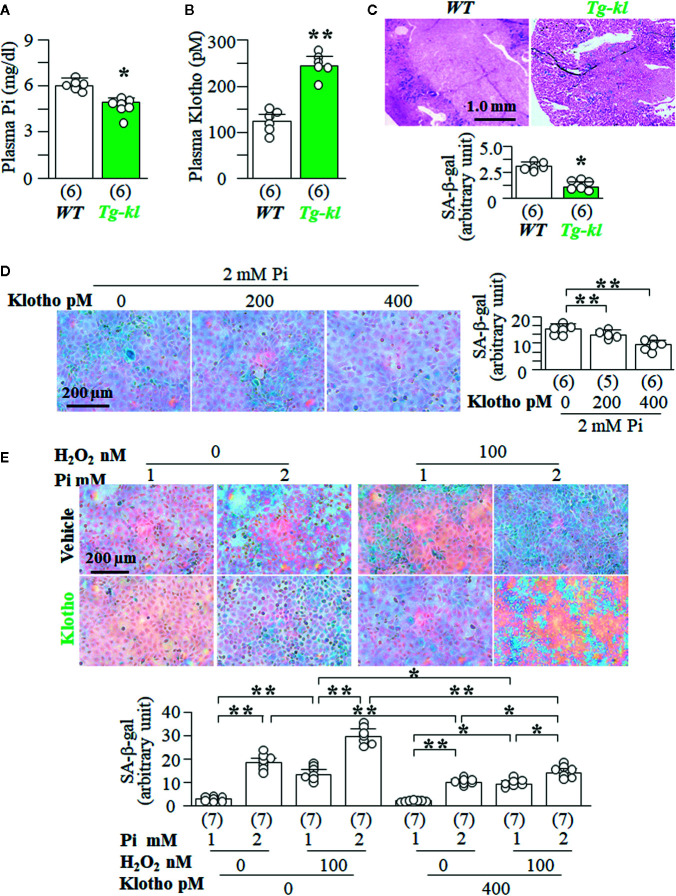
Klotho inhibits high Pi-induced senescence *in vivo* and *in vitro*. **(A–C)** *WT* mice and *Tg-kl* mice were maintained in normal Pi diet and sacrificed at 12 weeks old. Each genotype had 6 mice. **(A)** Plasma Cr. **(B)** plasma Klotho. **(C)** SA-β-gal stain in kidney sections of *WT* and *Tg-kl* mice. Upper panel: representative images of SA-β-gal stain; bottom panel: quantitative score of SA-β-gal stain. Data are presented as mean ± S.D. with scatter plots of individual data points. *P<0.05, **P<0.01 between two genotypes by un-paired Student t test for **(A–C)**. **(D, E)** NRK cells were seeded in 6-well plates and treated 2 mmol/l Pi with Klotho or vehicle (PBS) for 72 h. **(D)** SA-β-gal stain in NRK cells. Left panel: representative images of SA-β-gal stain; right panel: quantitative score of SA-β-gal stain. Sample number in each group is presented in bracket underneath corresponding bar. Data are presented as mean ± S.D. with scatter plots of individual data points. *P<0.05, **P<0.01 between two groups by one-way ANOVA followed by Student-Newman-Keuls *post hoc* test. **(E)** SA-β-gal stain in NRK cells treated with different levels of Pi or H_2_O_2_ or Klotho or vehicle. Upper panel: representative images of SA-β-gal stain; bottom panel: quantitative score of SA-β-gal stain. Sample number in each group is presented in bracket underneath corresponding bar. Data are presented as mean ± S.D. with scatter plots of individual data points. *P<0.05, **P<0.01 between two groups by three-way ANOVA followed by Student-Newman-Keuls *post hoc* test.

To explore the regulatory association of Klotho levels with senescence levels in the kidney, we used *Tg-kl* mice as *in vivo* model, because *Tg-kl* mice had lower levels of plasma Pi (**Figure 7A**) and higher levels of plasma Klotho (**Figure 7B**) than *WT* mice. Moreover, there was lower SA-β-gal staining score in the kidneys of *Tg-kl* mice compared to *WT* mice (**Figure 7C**), indicating lower senescence in the kidneys of *Tg-kl* mice. To examine the effect of Klotho on Pi-induced senescence, we co-treated NRK cells with recombinant Klotho protein and high Pi. We found that Klotho blunted Pi-induced senescence in a dose-dependent manner (**Figure 7D**). Furthermore, Klotho also effectively abolished synergistic effects of H_2_O_2_ and Pi on induction of senescence demonstrated by lower SA-β-gal staining (**Figure 7E**) and cell injury demonstrated by lower LDH and 8-OHdG (**Supplemental Figure 8**) compared to no Klotho incubation.

### Pi and Klotho Regulate Distinct Senescence Signaling Pathway in the Kidney

**Figure 8:**
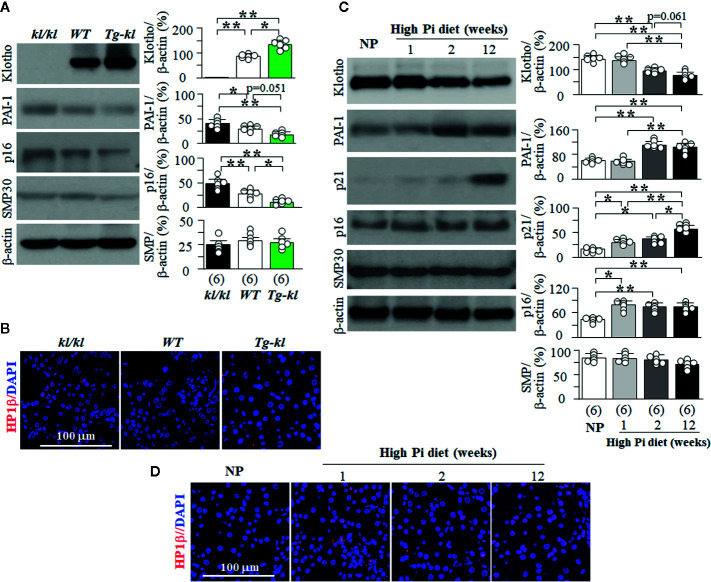
The changes in senescence signaling pathways induced by Pi and Klotho. **(A, B)** Three genotyping mice from low to high Klotho expression (*kl/kl, WT*, and *Tg-kl*) were maintained in normal Pi diet and sacrificed at 6 weeks old. Each genotype had 6 mice. **(A)** Immunoblots for Klotho and senescence markers in the kidneys. Left panel: representative immunoblots for Klotho, PAI-1, p16, SMP30, and β-actin (as loading control); right panel: summary of all immunoblots from each genotype. **(B)** Co-immunohistochemistry for senescence markers in kidney sections. Representative images for HP1β (red) and DAPI (blue) in kidney sections. **(C, D)** *WT* mice at 12 weeks old were fed with normal or high Pi diet for 1, 2, or 12 weeks, and sacrificed at assigned time points. Each Pi treatment had 6 mice. **(C)** Immunoblots for Klotho and senescence markers in total kidney lysates. Left panel: representative immunoblots for Klotho, PAI-1, p21, p16, SMP30, and β-actin; right panel: summary of all immunoblots from each group. **(D)** Immunohistochemistry for senescence markers in kidney sections. Representative microscopic images of co-immunostaining for HP1β (red) and DAPI (blue) in kidney sections. Sample number in each group is presented in bracket underneath corresponding bar. Data are presented as mean ± S.D. with scatter plots of individual data points. *P<0.05, **P<0.01 between any two groups by one-way ANOVA followed by Student-Newman-Keuls *post hoc* test for **(A, C)**.

Using murine models of genetic manipulation of the *Klotho* gene, we screened several key and well-known senescence signaling pathways in three genotypes ranging from Klotho deficiency to Klotho overexpression (*kl/kl, WT, Tg-kl*). There was a negative relationship between magnitude of Klotho protein expression and level of PAI-1 protein expression (**Figure 8A**). We also found higher p16 and p21 protein expression in the kidneys of *kl/kl* mice compared to *Tg-kl* mice (**Figures 8A, B**, and **Supplemental Figure 9**). These mice have simultaneous but reciprocal changes in Klotho and plasma Pi. To define the molecular signaling pathway whereby Pi modulates senescence, we next explored several key senescence proteins in the kidneys of *WT* mice treated with high Pi diet. The levels of plasma Pi started to mildly but significantly rise at 1 week of high dietary Pi and dramatically increased with decline in plasma Klotho at 2 weeks of high dietary Pi (**Supplemental Figure 10A**). Similarly, Klotho protein expression in the kidneys started to decrease at 2 weeks after high dietary Pi challenge (**Figure 8C**). Interestingly, the levels of p21 and p16 expression were appreciably upregulated starting 1 week after high Pi treatment (**Figure 8D** and **Supplemental Figure 10B**) suggesting that these changes might result from high Pi feeding and are independent of Klotho. PAI-1 and HP1β proteins were remarkably increased at 2 weeks after high dietary Pi while Klotho was decreased in the kidneys. This suggests that the changes in PAI-1 and HP1β in the kidney might be the result of either Klotho deficiency or the combination of both high Pi diet and Klotho deficiency.

The timeline of changes in Klotho and the markers of senescence in the kidneys (**Figures 8C, D**, and **Supplemental Figure 10B**) suggest that Pi may induce senescence through upregulation of p16 and p21, and Klotho deficiency might upregulate PAI-1 and HP1β to accelerate senescence. On the other hand, no change in SMP30 protein expression was detected in the kidney among the mice we studied (**Figures 8A, C**), suggesting that SMP30 is not involved in Pi and/or Klotho deficiency-associated senescence in the kidney.

### Klotho Suppresses SASP *In Vivo* and *In Vitro*

The senescence-associated secretory phenotype (SASP) encompasses numerous changes observed in senescent cells that secrete a variety of pro-inflammatory cytokines, chemokines, pro-fibrotic factors, immune modulators, growth factors, and proteases. Senescent cells function as the drivers and amplifiers to accelerate senescence through secretion and release of cytokines and factors in an autocrine and paracrine manner (Childs et al., 2015; Mchugh and Gil, 2018). SASP is a highly complex secretome with the precise composition varying markedly by cell and tissue context as well as the senescence-inducing stimuli.

**Figure 9:**
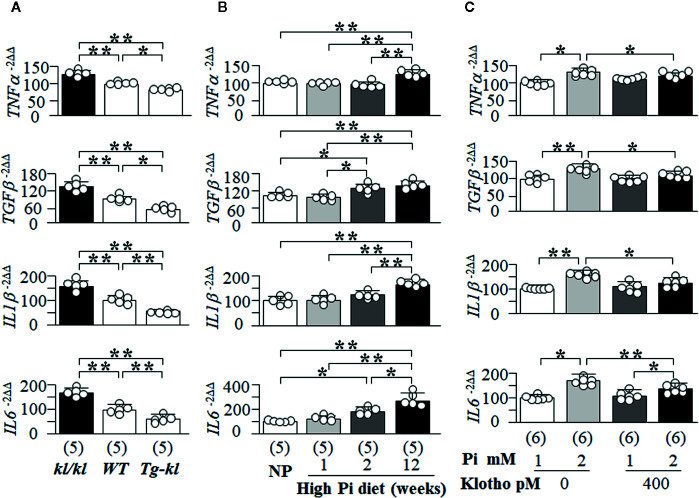
The changes in the senescence-associated secretory phenotype (SASP) regulated by Pi and Klotho. The levels of *TNFα*, *TGFβ*, *IL1β*, and *IL6* mRNA in the kidneys of mice were quantitatively analyzed with qPCR. **(A, B)**. **(A)** The changes in SASP among three genotyping mice from low to high Klotho expression (*kl/kl, WT*, and *Tg-kl*) at 6 weeks old. **(B)** The changes in SASP in *WT* mice treated normal diet or high Pi diet for 1, 2, and 12 weeks. Data are presented as mean ± S.D. with scatter plots of individual data points. Sample number in each group is presented in bracket underneath corresponding bar. *P<0.05, **P<0.01 between two groups by one-way ANOVA followed by Student-Newman-Keuls *post hoc* test. NRK cells were treated with 1 or 2 mM Pi, Klotho or PBS as control for 72 h and harvested for quantitative measurement of *TNFα*, *TGFβ*, *IL1β*, and *IL6* mRNA with qPCR **(C)**. Sample number in each group is presented in bracket underneath corresponding bar. Data are presented as mean ± S.D. with scatter plots of individual data points. *P<0.05, **P<0.01 between two groups by two-way ANOVA followed by Student-Newman-Keuls *post hoc* test.

Here, we first screened several key and well-known SASP-related cytokines and growth factors (TNFα, TGFβ, IL1β and IL6) in mice with different Klotho levels from genetic manipulation of Klotho gene. There were higher transcript levels of *TNFα*, *TGFβ*, *IL1β* and *IL6* in the kidneys of *kl/kl* mice compared to *Tg-kl* mice with *WT* mice being intermediate (**Figure 9A**), suggesting that Klotho deficiency or/and high Pi activates SASP in the kidneys because *kl/kl* mice have high plasma Pi and Klotho deficiency (**Figures 1B, C**, and **Supplemental Figure 4**) (Kuro-O et al., 1997).

To examine the effect of high Pi on SASP in the kidney, we examined SASP in mice fed with normal or high Pi diet and found that *TGFβ* and *IL6* mRNA were increased at 2 weeks after high dietary Pi challenge compared to normal Pi-fed mice (**Figure 9B**). But, the elevation of *TNFα* and *ILβ1* mRNA in the kidney transpired later (**Figure 9B**). Therefore, there is a heterogeneous response of changes in pro-inflammatory cytokines and pro-fibrotic factors to high Pi challenge.

After the high Pi diet, mice had higher plasma Pi and lower plasma and kidney Klotho (**Figure 8C** and **Supplemental Figure 10A**), which do not allow for the direct interrogation of Pi’s effect on SASP. To test for a direct effect, NRK cells were selected as the *in vitro* model. After incubation with high Pi for 2 days, the levels of *TNFα*, *TGFβ*, *IL1β* and *IL6* mRNA were significantly increased and elevation of *TNFα*, *TGFβ*, and *ILβ1* mRNA was dramatically blocked, and *IL6* partially blunted by recombinant Klotho protein (**Figure 9C**). Using H_2_O_2_-induced senescence in NRK cells (**Supplemental Figure 7** and **Figure 6**), which could be suppressed by recombinant Klotho protein (**Figure 7**), we tested if H_2_O_2_ could activate SASP and if Klotho could block this activation. Note that Klotho efficiently abrogated H_2_O_2_-activated SASP in NRK cells (**Supplemental Figure 11**).

### Senescence Is Directly Associated With Pi-Induced Cytotoxicity and EMT in NRK Cells

**Figure 10:**
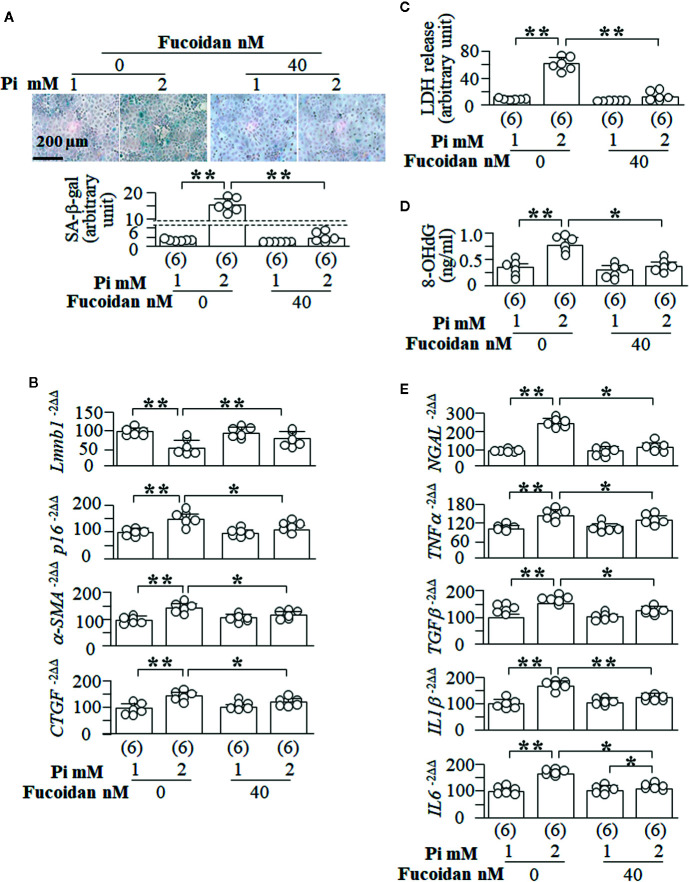
Senescence inhibitor blunts high Pi-induced cellular senescence, cytotoxicity and EMT *in vitro*. NRK cells were treated with fucoidan or vehicle for 72 h. After first 24 h, cells were treated with 1 or 2 mM Pi for 48 h. **(A)** SA-β-gal stain in NRK cells. Upper panel: representative images of SA-β-gal stain; bottom panel: quantitative score of SA-β-gal stain. Quantitative analysis of transcripts of **(B)** senescence markers (*Lmnb1* and *p16*) and fibrotic markers (*a-SMA* and *CTGF*) in the cells. **(C)** LDH and **(D)** 8-OHdG concentration in culture media from cultured NRK cells. **(E)** Quantitative analysis of transcripts of *NGAL* (a kidney tubular injury marker), *TNFα*, *TGFβ*, *IL1β*, and *IL6*. Sample number in each group is presented in bracket underneath corresponding bar. Data are presented as mean ± S.D. with scatter plots of individual data points. *P<0.05, **P<0.01 between two groups by two-way ANOVA followed by Student-Newman-Keuls *post hoc* test.

It is difficult to use *in vivo* animal models to prove the causal relation between senescence and cytotoxicity and fibrosis. We used fucoidan, a senescence inhibitor (Lee et al., 2015; Lee et al., 2018), to explore the role of the modulation of senescence in cytoprotection and fibrosis. We observed in NRK cells that fucoidan efficiently suppresses Pi-induced senescence demonstrated by the reduction of SA-β-gal score (**Figure 10A**) and *p16* mRNA as well elevation of *Lmnb1* mRNA (**Figure 10B**). Fucoidan also inhibited Pi-induced EMT as evident by reduction of α-SMA and CTGF (**Figure 10B**). Pi-induced cell injury (LDH release) (**Figure 10C**), oxidative DNA damage (8-OHdG determination) (**Figure 10D** **)** and NGAL expression (**Figure 10E**) in NRK cells were also significantly improved, suggesting that fucoidan protect cells against phosphotoxicity. Similar to Klotho (**Supplemental Figures 8** and **11**), Fucoidan also significantly attenuated SASP activation induced by high Pi (**Figure 10E**). Therefore both prevent senescence amplification and oxidative stress in the kidney.

## Discussion

**Figure 11:**
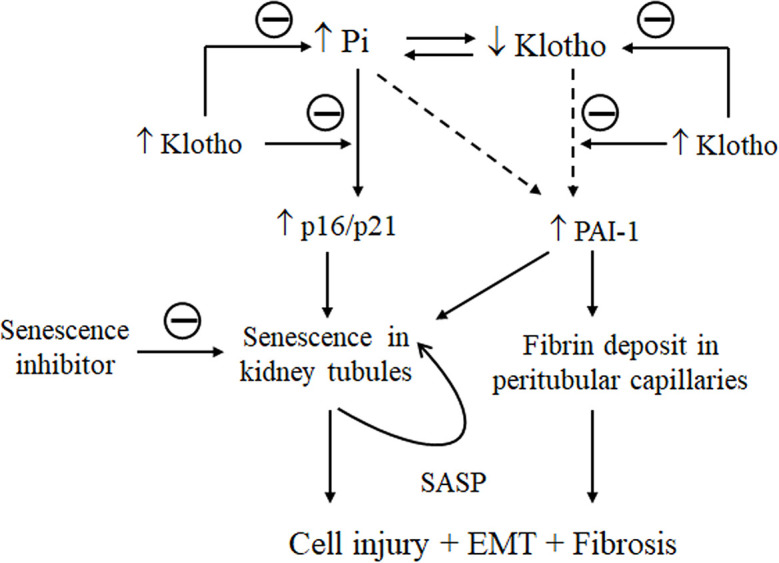
Schema for the effect of Klotho and Pi on senescence and fibrosis in the kidney. On the one hand, high Pi upregulates p16 and p21 expression in the kidney and induces senescence (early phase), PAI-1 upregulation, and Klotho downregulation (later phase). Whether Pi stimulates PAI-1 dependently or independently of upregulation of p16/p21 or downregulation of Klotho signaling is unclear (dash line). High PAI-1 promotes fibrin deposits in peritubular capillaries, and also activates senescence, injury and EMT in kidney tubules. On the other hand, Klotho deficiency increases plasma Pi. Senescence amplifies the number of senescent cells through activation of a vicious cycle of secreted pro-inflammatory cytokines and pro-fibrotic growth factors called SASP. As a result, elevation of senescence in kidney tubules and massive deposits of fibrin in vessels trigger or/and accelerate fibrogenesis in the kidney. Klotho could improve Pi homeostasis, attenuate the upregulation of p16/p21 and PAI-1 to decrease senescence, and alleviate kidney fibrosis.

Senescence is a phenomenon by which normal cells stop dividing after exposure to a variety of stimuli. We showed here that high Pi loading, oxidative stress, and Klotho deficiency individually and synergistically stimulate senescence. Increased senescence in the kidney associated with kidney fibrosis is present in high Pi-fed mice and in CKD mice. High Pi enhanced H_2_O_2-_induced cellular senescence and injury. Suppression of Pi-activated senescence in NRK cells by Klotho and Fucoidan, a senescence inhibitor, effectively attenuated cell injury and EMT induced by Pi or/and H_2_O_2_. Based on the timeline of changes in p16/p21 and PAI-1 expression, plasma Pi and kidney Klotho levels in mice treated with high dietary Pi together with results in cultured cells treated with high Pi media, we propose that Pi first activates p16/p21 signaling followed by upregulation of PAI-1 and downregulation of Klotho. Klotho is a promising strategy to protect against kidney aging and fibrosis through reduction of senescence (**Figure 11**).

### High Pi and Low Klotho Are Associated With High Senescence in the Kidney

CKD is interpreted as a form of premature or accelerated aging (Stenvinkel and Larsson, 2013; Kooman et al., 2014). High senescence in kidney tubules of CKD animals (Luo et al., 2018; Knoppert et al., 2019) can be activated by uremic serum (Buendia et al., 2015), indoxyl sulfate (Shimizu et al., 2010; Niwa and Shimizu, 2012), oxidative stress (Adijiang and Niwa, 2010), and Wnts (Luo et al., 2018). The current study indicated that high senescence in kidney of CKD mice can be induced by high Pi and low Klotho. We further found that normal mice had modest increase in plasma Pi with relatively normal plasma Klotho, but had significantly high expression of p16/p21 in the kidneys at 1 week after high dietary Pi treatment, indicating that high Pi activates kidney senescence independently of Klotho, which was supported by *in vivo* results that senescence markers were upregulated by high Pi diet, followed by downregulation of kidney Klotho, and *in vitro* results that high Pi media induced cellular senescence, injury and EMT in NRK cells lacking endogenous Klotho expression. This is a proof-of-concept study, and the cellular and molecular mechanisms of Pi effect on senescence are not illustrated. Whether pathologic actions of Pi are mediated by increase in intracellular Pi through Pi uptake by Pi transporters (Moe, 2009; Villa-Bellosta et al., 2009; Wallingford and Giachelli, 2014; Yamada et al., 2018; Levi et al., 2019) of type III Na-Pi co-transporters and/or by activation of yet-to-be determined intracellular signaling through Pi transport-independent activity (Beck et al., 2009) remains to be studied.

On the other hand, phosphate regulation of Klotho expression in the kidney was reported nearly 20 years ago (Morishita et al., 2001). We (Hu et al., 2015; Shi et al., 2018b; Hu et al., 2019; Shi et al., 2020) and other investigators (Morishita et al., 2001; Perwad et al., 2005; Ohnishi and Razzaque, 2010; Watanabe et al., 2017; Yoshikawa et al., 2018; Fukuda-Tatano et al., 2019) devoted efforts to study the effect of Pi on Klotho expression and (intra)cellular mechanisms of phosphate toxicity, but the answer is still elusive. We found that high Pi induced secretion of cytokines including TNFα and TGFβ (**Figure 9**), induced oxidative stress (**Figures 6** and **10**, **Supplemental Figures 7** and **8**). All these factors (Mitobe et al., 2005; Thurston et al., 2010; Moreno et al., 2011; Zhou et al., 2013) released from proximal tubules may contribute to reduction of Klotho in adjacent distal tubules through paracrine manner. Based on the time line of the changes in senescence markers, oxidative stress, SASP (release of cytokines including TNFα and TGFβ), and Klotho expression, we proposed that high Pi stimulates senescence strongly in proximal tubules and weakly in distal tubules, induces oxidative stress, and activates SASP, and consequently downregulating Klotho expression in distal tubules.

Furthermore, the cytoprotective effect of Fucoidan on Pi-induced senescence and cell injury in NRK cells suggests the role of Pi in the pathogenesis of senescence and cellular plasticity. However, we and other investigator showed that Fucoidan is a compound with multiple biologic functions including anti-oxidation, the possibility that Fucoidan inhibition of senescence is associated with anti-oxidation could not be excluded. In the future, we need to conduct an *in vivo* experiment to confirm beneficial effects of Fucoidan on Pi-activated senescence and kidney fibrosis.

Senescent cells are prominent in PT and TAL in the cortex, and weakly in DT and CD in the outer medulla in the kidney of *WT* mice. This pattern is consistent with the fact that these kidney segments are the most susceptible to kidney insults (Portilla, 2014; Kefaloyianni et al., 2016; Kramann et al., 2017) and putative sites of EMT (Liu, 2004; Portilla, 2014; Gewin, 2018). Therefore, senescence in kidney tubules may be one of the mediator of cellular injury and plasticity of kidney epithelial cells after exposure to kidney insults (Kang and Johnson, 2003; Basile, 2007; Meran and Steadman, 2011; Humphreys, 2018).

### High Pi and Low Klotho Are Associated With High PAI-1 in the Kidney

Klotho deficiency is associated with kidney fibrosis and mediated by abnormal Wnt (Luo et al., 2018) and TGFβ signaling pathways (Doi et al., 2011). Additionally, PAI-1 over-expression is associated with kidney fibrosis and fibrin deposition and senescence in Klotho-deficient mice (Takeshita et al., 2002; Eren et al., 2014). The current study clearly showed fibrin deposition in peritubular capillaries of *kl/kl* mice. Chronic fibrin deposition and accumulation destroys kidney tubules and stimulates fibrosis. PAI-1 is a well-known key player of fibrinolysis (Hekman and Loskutoff, 1987), and also considered as a marker and a mediator of senescence (Takeshita et al., 2002; Eren et al., 2014; Vaughan et al., 2017). Increase in PAI-1 in NRK cells treated with high Pi media suggests that Pi is an inducer of PAI-1 expression. The *in vivo* observation that high Pi induces p16/p21 activation first followed by upregulation of PAI-1 and downregulation of Klotho provides an alternative mechanism by which PAI-1 may be induced by low Klotho or high p16/p21 signaling activity.

It has been shown that in *kl/kl* hypomorphic mice, vascular endothelial growth factor (VEGF)-mediated internalization of the VEGF receptor-2 (VEGFR-2/transient receptor potential cationic channel subfamily C-1 (TRPC-1) complex is impaired, and VEGF-mediated elevation of Ca^2+^ was sustainably high. Thus endothelial integrity is broken and vessel permeability is increased, which can be reversed by Klotho (Kusaba et al., 2010). As a consequence, massive fibrin deposition in vessels and in adjacent tubules and interstitium stimulates fibrogenesis and contributes kidney fibrosis in *kl/kl* mice. Whether excessive fibrin deposition can activate senescence is yet to be determined.

SMP30 is abundantly expressed in kidney proximal tubule cells, and its expression decreases with age. Loss of SMP30 is associated with kidney damage in several mouse models (Yumura et al., 2006; Kondo et al., 2013; Miyata et al., 2013; Okada et al., 2015; Kondo and Ishigami, 2016; Yamauchi et al., 2016; Dutta et al., 2019). However, we did not find reduced SMP30 expression in mouse kidney and NRK cells. The discrepancy between our results and published data indicates that Pi-activated senescence is independent from SMP30.

### Senescence Is a Potential Target for Protection of Kidney Tubules and Prevention Against Kidney Fibrosis

Klotho was shown to inhibit senescence in the kidney of uremic animals (Adijiang and Niwa, 2010), Klotho-deficient mice (Eren et al., 2014) and in several cell lines (De Oliveira, 2006; Ikushima et al., 2006). It seems that intracellular, rather the secreted form of Klotho interacts with retinoic-acid-inducible gene-I to inhibit SASP and inflammation (Liu et al., 2011). However, many studies confirmed secreted Klotho also inhibits senescence de *in vivo* (Chen et al., 2018) and *in vitro* (Kuro-O, 2008). Our *in vitro* data supports the protective effect of soluble Klotho on suppression of cellular senescence, oxidation, injury and EMT in NRK cells induced by Pi. Moreover, Pi exacerbated H_2_O_2_-activated kidney senescence and injury, which was attenuated by soluble Klotho. Therefore, soluble Klotho might be a promising strategy for senescence-associated disease.

The current study also clearly showed that high Pi induces normal cells to become senescent cells, which secret TNFα, TGFβ, IL1β and IL6. Based on the order of changes in an increase in senescence markers, TGFβ expression and fibrotic markers in mice fed high Pi diet, we proposed that high Pi induced cells to develop senescence, and subsequently senescent cells secrete profibrotic growth factors including TGFβ, kidney fibrosis develops through EMT. However, whether high Pi activates resident fibroblasts in the kidney or induces endothelial mesenchymal transition and hence causing kidney fibrosis remains to be determined. High SASP activation renders adjacent normal cells senescent in a paracrine manner, and amplifies inflammation and EMT, hence promoting CKD progression. Klotho overexpressing mice had low SASP activation, and soluble Klotho suppressed Pi- and H_2_O_2_-activated SASP, similar to Fucoidan’s effect, supporting that Klotho is a senescence suppressor. Given that high Pi activates cellular senescence in the kidney, whether low Pi reduces cellular senescence or synergistically enhances senescence inhibitor in kidney disease still needs to be determined.

### In Conclusion

High Pi induces senescence through upregulation of p16/p21 and PAI-1 signaling, which is amplified by oxidative stress and suppressed by Klotho. In addition to maintenance of Pi homeostasis, Klotho, as an endogenous senescence inhibitor like Fucoidan, protects cells from oxidative injury and Pi toxicity, and suppresses EMT in cultured kidney tubular cells exposed to Pi or/and H_2_O_2_. Therefore, Klotho is a promising senescence inhibitor to protect the kidney from Pi toxicity and suppress kidney fibrosis.

## Data Availability Statement

All datasets presented in this study are included in the article/**Supplementary Material**.

## Ethics Statement

The animal study was reviewed and approved by UT Southwestern Medical Center, IACUC.

## Author Contributions

OM and MH conceived the experiments and designed the study. JM, BF, MS, SS, ZZ, and SY carried out the experiments. JM, BF, MS, SS, SY, and MH made the figures. JM, MS, SY, OM, and MH analyzed data. JM, MS, and MH drafted the manuscript. OM and MH revised the manuscript. All authors contributed to the article and approved the submitted version.

## Funding

The work in authors’ research laboratories was supported in part by NIH R01 grants DK091392 and DK092461 (OM. and MH), the O’Brien Kidney Research Center at UT Southwestern (P30-DK07938) (OM), the Pak Center Innovative Research Support, Endowed Professors Collaborative Research Support, and the Pak-Seldin Center for Metabolic Research (OM and MH).

## Conflict of Interest

The authors declare that the research was conducted in the absence of any commercial or financial relationships that could be construed as a potential conflict of interest.
