# Supplementary material for: High Phosphate Induces and Klotho Attenuates Kidney Epithelial Senescence and Fibrosis
Source: Front Pharmacol. 2020 Aug 20;11:1273. doi: 10.3389/fphar.2020.01273 (PMC7468469; doi:10.3389/fphar.2020.01273)
Supplement: Supplementary file 1 [file DataSheet_1.pdf]

High phosphate induces and Klotho attenuates kidney epithelial senescence and fibrosis

Jenny Maique[1], Brianna Flores[1], Mingjun Shi[1], Sierra Shepard[1], Zhiyong Zhou[1], Shirely Yan[3], Orson W. Moe[1,2,4], Ming Chang Hu[1,2,*]

[1]Charles and Jane Pak Center for Mineral Metabolism and Clinical Research
Departments of [2]Internal Medicine, [3]Pathology, and [4]Physiology
University of Texas Southwestern Medical Center, Dallas, TX, USA

Running title: Klotho and phosphate in senescence and kidney fibrosis

*** Correspondence:**

Ming Chang Hu, M.D.; Ph.D.
Charles and Jane Pak Center for Mineral Metabolism and Clinical Research
Department of Internal Medicine
The University of Texas Southwestern Medical Center
5323 Harry Hines Blvd., Dallas, TX 75390 USA
Email: ming-chang.hu@utsouthwestern.edu

SUPPLEMENTAL FIGURE LEGENDS

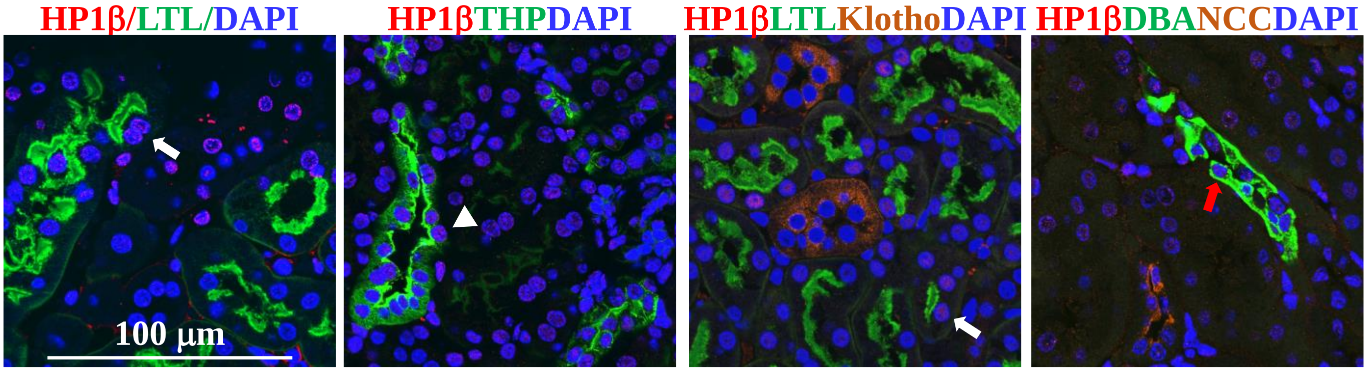

Supplemental Figure 1 Co-immunostaining for HP1β (red) with different kidney tubular markers and DAPI for nuclear stain (blue) in the kidneys of *WT* mice at 12 weeks old. Immunostaining was performed in at least three independent experiments showing similar results. **Far left panel**: Representative images of HP1β, LTL (marker for proximal tubule-PT, green) and DAPI; **middle left panel**: Representative images of HP1β, THP (marker for thick ascending limb of loop of Henle-TAL, green) and DAPI; **middle right panel**: Representative images of HP1β, LTL (marker for PT, green); Klotho (marker for distal tubule-DT, brown) and DAPI; and **far right panel**: Representative images of HP1β, DBA (marker for collecting duct-CD, green), NCC (marker for DT, brown) and DAPI. White arrows depict HP1β in PT, white arrow head in TAL, and red arrow in CD.

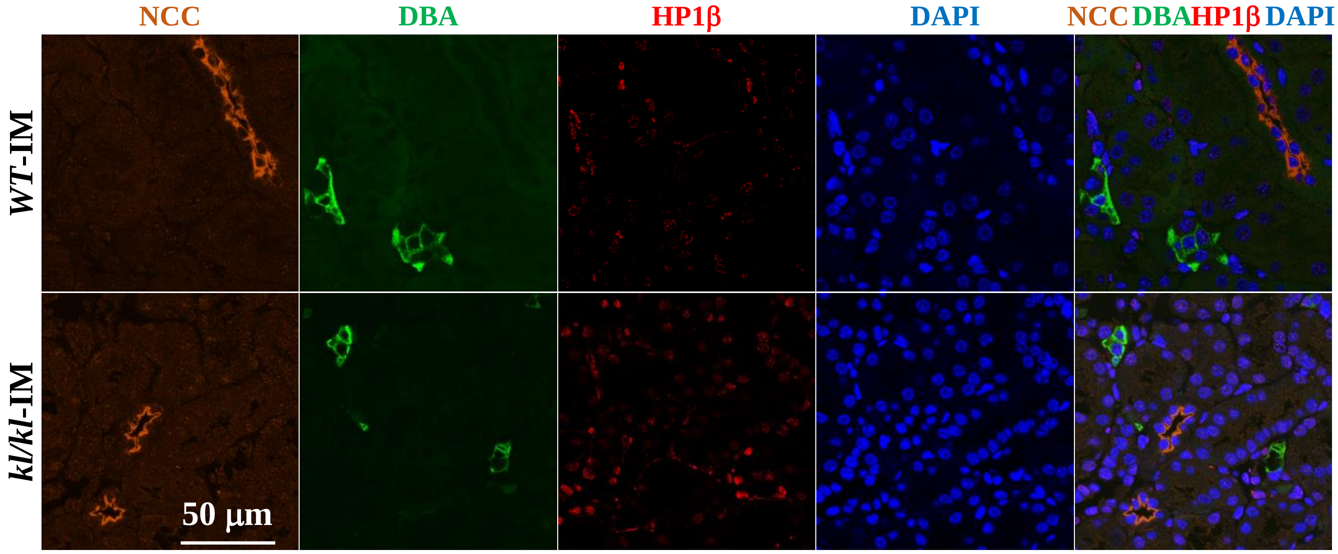

Supplemental Figure 2 Co-localization of HP1β (red) in distal tubules stained with NCC (brown), collecting ducts stained with DBA (green) in inner medulla (IM) of kidneys from *WT* and *kl/kl* mice at 6-week old. DAPI (blue): nuclear stain.

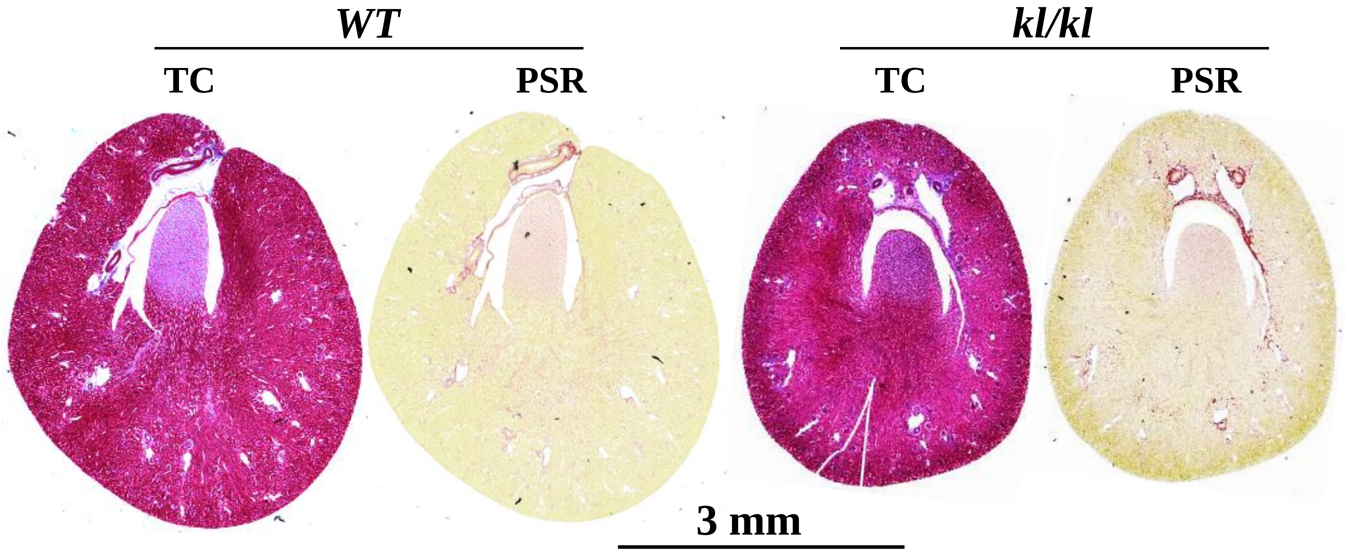

Supplemental Figure 3 Massive tubulointerstitial fibrosis in *kl/kl* mice compared to *WT* mice fed with normal Pi diet and sacrificed at 6 weeks old. Trichrome (TC) stain and picro-sirius red (PSR) stain in kidney sections of *WT* mice (**left panel**) and *kl/kl* mice (**right panel**).

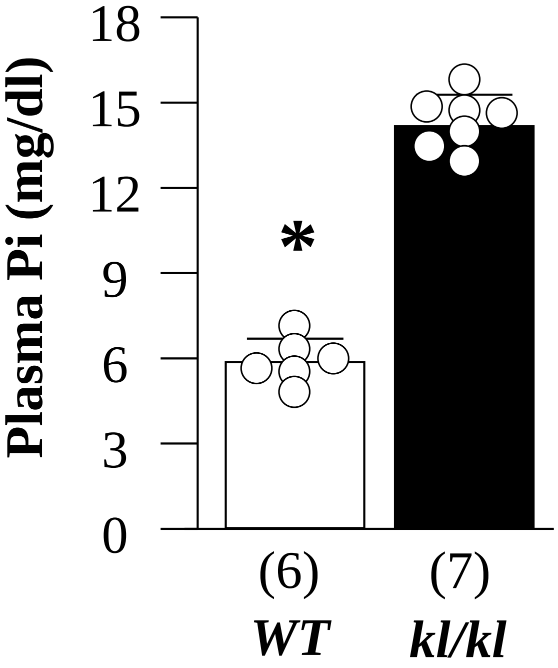

Supplemental Figure 4 High plasma Pi in *kl/kl* mice than in *WT* fed with normal Pi diet at 6 weeks old. Sample number in each group is shown in bracket underneath corresponding bar. Data are presented as mean ± S.D. with scatter plots of individual data points. *P<0.05, **P<0.01 between two genotypes by un-paired Student t test.

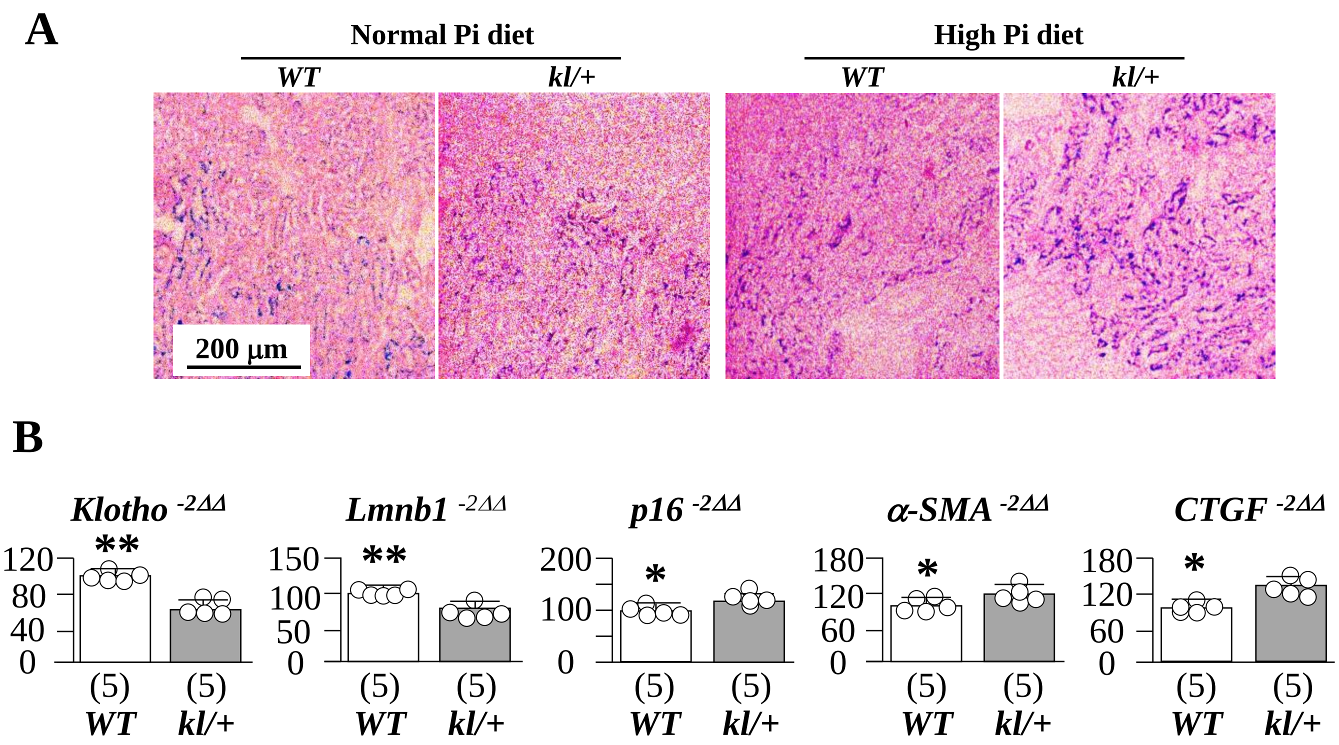

Supplemental Figure 5 *WT* mice and *kl/+* mice were fed with normal or high dietary phosphate chow for 12 weeks. (**A**) SA-β-gal stain was performed in the kidney sections. Representative microscopic images of SA-β-gal stain. (**B**) Quantitative analysis of transcripts of *Klotho*, senescence markers (*Lmnb1* and *p16*), and fibrotic markers (*a-SMA*, and *CTGF*) in the kidneys of *WT* and *kl/+* mice at 12 weeks old were fed with 12-week high phosphate diet. Sample number in each group is presented in bracket underneath corresponding bar. Data are presented as mean ± S.D. with scatter plots of individual data points. *P<0.05, **P<0.01 between two genotypes by un-paired Student t test.

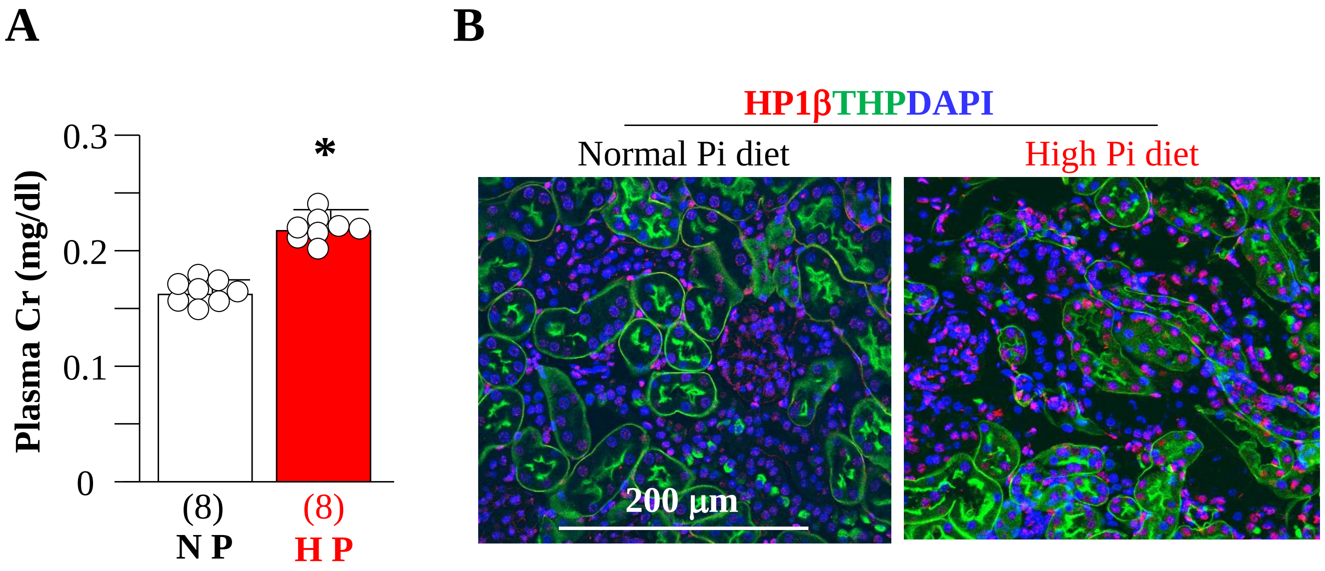

Supplemental Figure 6 High Pi promotes cellular senescence in the kidney and accelerates kidney deterioration in CKD mice. *WT* mice at 12 weeks old underwent to CKD induction surgery and were fed with normal or high Pi diet for 12 weeks starting 2 weeks after CKD induction. (**A**) Plasma Cr. Sample number in each group is shown in bracket underneath corresponding bar. Data represented as mean ± S.D. with scatter plots of individual data points. *P<0.05 between two genotypes by un-paired Student t test. (**B**) Immunohistochemistry for HP1β (red), THP (TAL marker, green), and DAPI (blue) in the kidney sections.

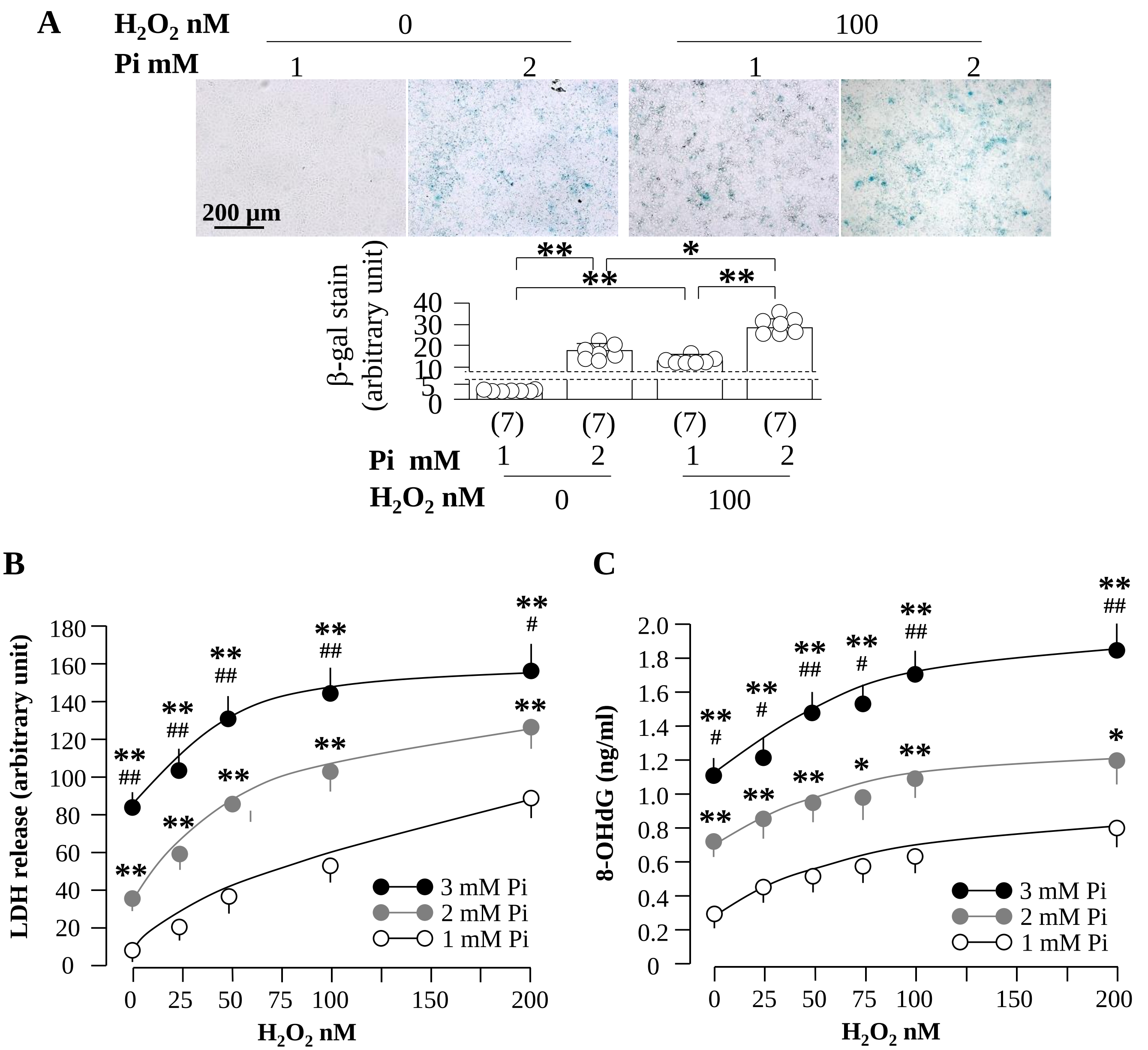

Supplemental Figure 7 High Pi exacerbates H_2O_2-induced senescence and cell injury. NRK cells were seeded in 6-well plates and treated with Pi for 72 hours. After 24 hours of Pi incubation, the cells were treated with H_2O_2 for 48 hours. (**A**) SA-β-gal stain in the cells with eosin as the counter stain.. **Upper panel**: Representative images of SA-β-gal stain; **bottom panel**: Quantitative score of SA-β-gal stain. Sample number in each group is presented in bracket underneath corresponding bar. Data are presented as mean ± S.D. with scatter plots of individual data points. *P<0.05, **P<0.01 between two groups by two-way ANOVA followed by Student-Newman-Keuls *post hoc* test. (**B**) LDH and (**C**) 8-OHdG concentration in cultured media collected from NRK cells treated with Pi or/and H_2O_2. Each time point had seven samples. Data are presented as mean ± S.D. *P<0.05, **P<0.01 vs 1 mM Pi; $^{\#}$P<0.05 vs 2 mM Pi at same dose of H_2O_2 by one-way ANOVA followed by Student-Newman-Keuls *post hoc* test.

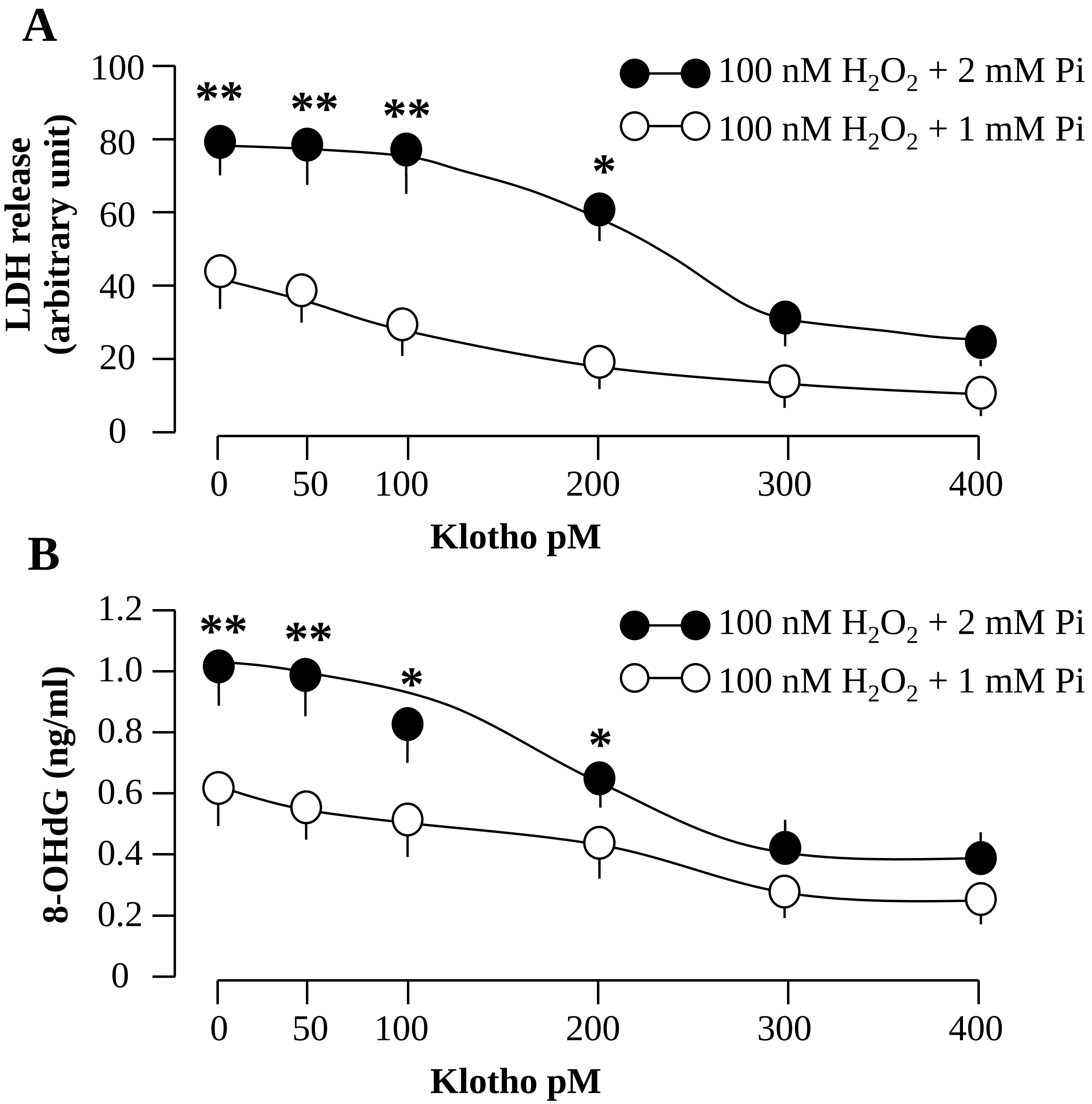

Supplemental Figure 8 Klotho inhibits high Pi-induced senescence *in vitro*. NRK cells were seeded in 6-well plates and treated with 2 mmol/l Pi, Klotho or vehicle (PBS) for 72 hours. (**A**) LDH and (**B**) 8-OHdG concentration in cultured media collected from NRK cells. Each time point has seven samples. Data are presented as mean ± S.D. *P<0.05, **P<0.01 between 100 nM H_2O_2 + 1 mM Pi and 100 nM H_2O_2 + 2 mM Pi at same dose of Klotho by unpaired Student t test.

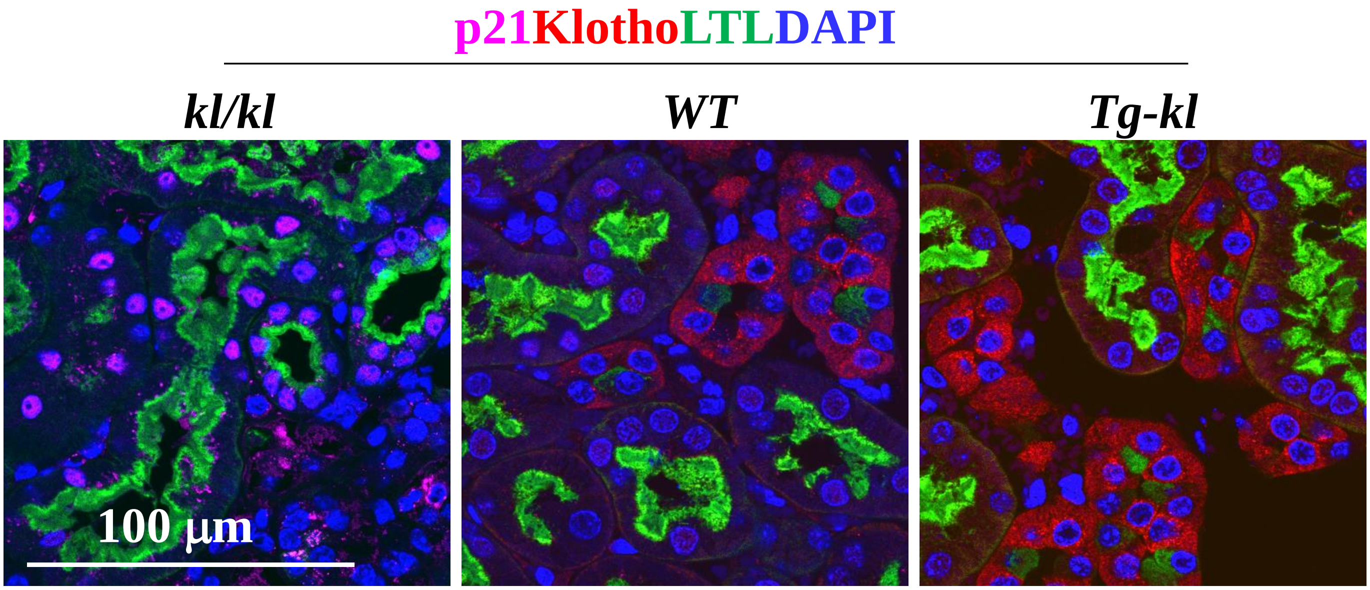

Supplemental Figure 9 The senescence in the kidneys of *kl/kl*, *WT*, and *Tg-kl* fed with normal Pi diet at 6 weeks old. Co-immunohistochemistry for senescence markers in kidney sections. Representative images for p21 (pink), Klotho (red), LTL (green), and DAPI (blue) in kidney sections..

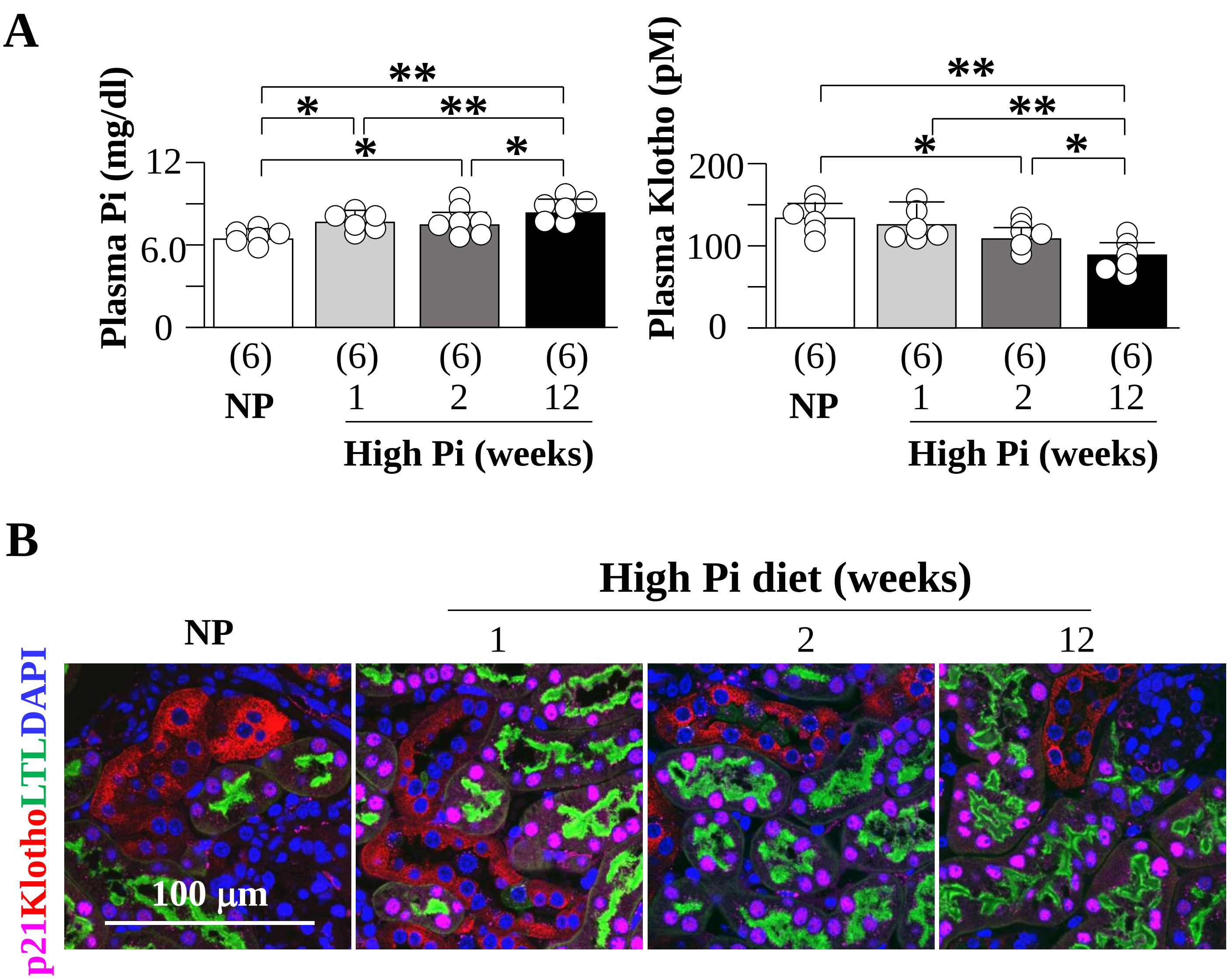

Supplemental Figure 10 High Pi diet increases plasma Pi and decreases plasma Klotho. *WT* mice at 12 weeks old were fed with normal or high Pi diet for 1, 2 or 12 weeks. (**A**) Plasma Pi (**left panel**) and Klotho (**right panel)**). Sample number in each group is shown in bracket underneath corresponding bar. Data are presented as mean ± S.D. with scatter plots of individual data points. *P<0.05, **P<0.01 between any two groups by one-way ANOVA followed by Student-Newman-Keuls *post hoc* test. (**B**) Representative microscopic images of co-immunostaining for p21 (pink), Klotho (red), LTL (green), and DAPI (blue) in kidney sections of *WT* mice fed with different Pi diets.

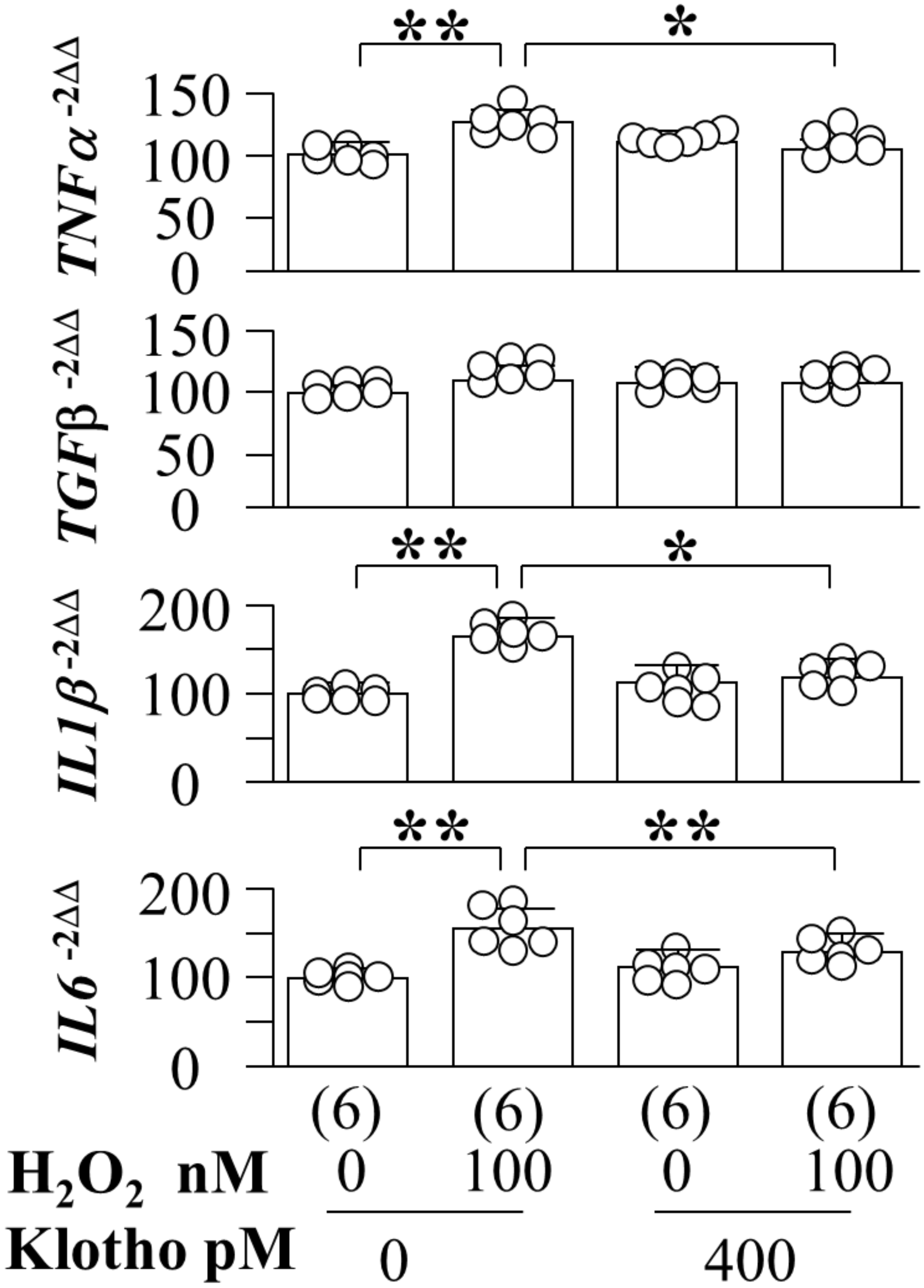

Supplemental Figure 11 Pi and Klotho regulate the senescence-associated secretory phenotype (SASP). NRK cells were treated with Pi, H_2O_2, or Klotho for 48 hours, and harvested for quantitative measurement of *TNF*α, *TGF*β, *IL1*β, and *IL6* mRNA with qPCR. Sample number in each group is presented in bracket underneath corresponding bar. Data are presented as mean ± S.D. with scatter plots of individual data points. *P<0.05, **P<0.01 between two groups by two-way ANOVA followed by Student-Newman-Keuls *post hoc* test.
